# The CPEB translational regulator, Orb, functions together with Par proteins to polarize the *Drosophila* oocyte

**DOI:** 10.1371/journal.pgen.1008012

**Published:** 2019-03-13

**Authors:** Justinn Barr, Sofia Charania, Rudolf Gilmutdinov, Konstantin Yakovlev, Yulii Shidlovskii, Paul Schedl

**Affiliations:** 1 Department of Molecular Biology, Princeton University, Princeton, New Jersey, United States of America; 2 Laboratory of Gene Expression Regulation in Development, Institute of Gene Biology, Russian Academy of Sciences, Moscow, Russia; University of Cambridge, UNITED KINGDOM

## Abstract

*orb* is a founding member of the CPEB family of translational regulators and is required at multiple steps during Drosophila oogenesis. Previous studies showed that *orb* is required during mid-oogenesis for the translation of the posterior/germline determinant *oskar* mRNA and the dorsal-ventral determinant *gurken* mRNA. Here, we report that *orb* also functions upstream of these axes determinants in the polarization of the microtubule network (MT). Prior to *oskar* and *gurken* translational activation, the oocyte MT network is repolarized. The MT organizing center at the oocyte posterior is disassembled, and a new MT network is established at the oocyte anterior. Repolarization depends upon cross-regulatory interactions between anterior (apical) and posterior (basal) Par proteins. We show that repolarization of the oocyte also requires *orb* and that *orb* is needed for the proper functioning of the Par proteins. *orb* interacts genetically with *aPKC* and *cdc42* and in egg chambers compromised for *orb* activity, Par-1 and aPKC protein and *aPKC* mRNA are mislocalized. Moreover, like *cdc42*^*-*^, the defects in Par protein localization appear to be connected to abnormalities in the cortical actin cytoskeleton. These abnormalities also disrupt the localization of the spectraplakin Shot and the microtubule minus-end binding protein Patronin. These two proteins play a critical role in the repolarization of the MT network.

## Author summary

The specification of polarity axes in the *Drosophila* egg and embryo depends on the proper organization of the microtubule (MT) and actin cytoskeleton during mid-oogenesis. During this period, the MT organizing center at the posterior of the oocyte is disassembled and a MT network is established at the anterior and anterior-lateral cortex of the oocyte. We show that the CPEB translation factor *orb* plays a critical role in the reorganization of the MT network. *orb* appears to function at several levels during MT reorganization. *orb* interacts genetically with genes encoding Par proteins, *aPKC* and *cdc42*, and disrupts the localization of Par-1 and aPKC within the oocyte. *orb* also plays an important role in organizing the cortical actin cytoskeleton. The defects in the actin cytoskeleton disrupt the cortical association of Shot and Patronin, which are responsible for nucleating the assembly of the anterior MT network.

## Introduction

Specification of the anterior-posterior (AP) and dorsal-ventral (DV) axes of the *Drosophila* embryo depends upon determinants that are localized within the egg during oogenesis [1–4]. For example, expression of the TGF-α cell signaling molecule Gurken (Grk) at the anterior corner of the oocyte during mid-to-late oogenesis establishes the DV axis of the egg and subsequently the embryo by signaling to the overlying somatic follicle cells [5–8]. Factors important in determining the AP axis of the embryo are also localized during this same period. Specification of the posterior axis is mediated by *oskar* (*osk*) [9, 10]. *osk* mRNA is targeted to the posterior cortex of the oocyte, where it is translated and functions in the assembly of the pole plasm and the anchoring of the mRNA encoding the posterior determinant *nanos* [11, 12]. The anterior axis is specified by the Bicoid transcription factor, and its mRNA is localized to the anterior cortex of the oocyte [13–15].

The proper localization of these determinants within the oocyte during mid-to-late oogenesis depends upon the disassembly of the existing microtubule cytoskeleton (MT) during stage 7 of oogenesis and its subsequent repolarization [7, 8]. The polarity of the MT network in the period prior to stage 7 is established early in oogenesis when the oocyte is initially specified [16]. A microtubule organizing center (MTOC) is assembled at the oocyte cortex just posterior to the oocyte nucleus and it directs the elaboration of the MT network by anchoring the minus-ends of MTs. As a consequence of this polarization of the oocyte, mRNAs encoding determinants critical for the early stages (stage 1–7) of egg chamber development accumulate at the posterior cortex. One of these is *gurken* (*grk*) mRNA. Grk protein translated from this localized message signals to the somatic follicle cells covering the posterior of the egg chamber to specify posterior follicle cell fate (PFC) [7, 8]. Subsequently, during stage 7, an unknown signal(s) emanating from the somatic PFCs triggers the repolarization of MT network in the germline. This signal induces the disassembly of the posterior MTOC and the network of MTs extending from the MTOC towards the anterior of the oocyte [7, 8, 17–20]. At the same time, *de novo* MT assembly is nucleated along the anterior and lateral cortex of the oocyte by a centrosome independent mechanism. This mechanism deploys the tubulin minus-end binding protein Patronin and the actin-MT linker Short Stop (Shot) [21]. Accompanying the repolarization of the MT cytoskeleton, the oocyte nucleus migrates from the posterior end of the oocyte to the anterior corner [22]. *grk* mRNA also relocates so that it is positioned between the oocyte nucleus and the oocyte cortex. Grk protein expressed from the localized message signals dorsal follicle cell fate and this defines the DV axis of the egg chamber and embryo [6, 23].

In addition to Patronin and Shot, the other factors implicated in oocyte repolarization are the *Drosophila* homologs of the *par*tioning-defective (Par) group genes, *par-1*, *cdc42* and *bazooka* (*baz*/*par*-*3*) [24–26]. These three genes together with *par*-*6* and *aPKC* are also required for the initial polarization of the stage 1 egg chamber [24–30]. These proteins generate cellular asymmetries by inhibitory cross-regulatory interactions that impede association with the cell cortex [25, 31–33]. During MT repolarization, Par-1 becomes enriched along the posterior cortex of the oocyte [34–36]. There is a complementary distribution of Baz, Par-6, aPKC and Cdc42: they are enriched along anterior and anterior-lateral cortex, but not the posterior [24, 25, 37, 38]. The available evidence indicates that the asymmetry in the oocyte generated by the activation of the Par polarity network is upstream of the localization of Shot and Patronin along the anterior and lateral cortex, and thus the Patronin dependent *de novo* assembly of MTs [21].

In addition to being critical for properly localizing *grk*, *osk* and *bcd* mRNAs, the reorganization of the cytoskeleton also alters the distribution of other mRNAs encoding oocyte-specific proteins. One of these mRNAs is *orb*, which encodes one of the two fly cytoplasmic polyadenylation element RNA-binding (CPEB) proteins [39, 40]. During early stages of oogenesis, *orb* mRNA is localized at the posterior of the oocyte. After repolarization *orb* mRNA disappears from the posterior and becomes concentrated along the anterior-lateral margin of the oocyte [41]. While the rearrangement of *orb* mRNA within the oocyte is clearly downstream of the steps involved in repolarizing the oocyte MT network, the *orb* gene plays a central role in the initial formation and subsequent development of the oocyte and thus could be an active participant in determining oocyte polarity.

In ovaries, *orb* expression is restricted to the germline and is required at multiple steps during oogenesis [39, 40, 42]. In wild type ovaries, a cystoblast, generated by an asymmetric division of a stem cell, undergoes four mitotic divisions with incomplete cytokinesis to produce a 16-cell cyst [1]. In the *orb* null allele, *orb*^*343*^, the last of these mitotic divisions is not completed and the cyst degenerates [40]. While the strong loss-of-function allele, *orb*^*303*^, forms a 16-cell cyst, the oocyte is not properly specified and egg chambers contain only nurse cells [40]. Unlike *orb*^*343*^ and *orb*^*303*^, the Orb protein expressed by the hypomorphic *orb* allele, *orb*^*mel*^, is wild type. Instead, *orb*^*mel*^ transcripts are incorrectly spliced generating an mRNA lacking sequences from the 5’UTR [42]. The removal of these 5’ sequences alters Orb expression as oogenesis proceeds. Prior to stage 7 the level and localization of Orb in the oocyte is similar to that observed in wild type. However, beginning around stage 7, the amount of Orb drops dramatically and most chambers have little residual protein. As a consequence of this reduction in Orb protein, *orb*^*mel*^ females produce eggs that give rise to embryos with a range of phenotypic abnormalities including D-V and A-P patterning defects [42]. These patterning defects arise from a failure in the localization and/or translation of two Orb regulatory targets, *grk* and *osk* mRNAs, during mid-to-late oogenesis [43–46].

*grk* and *osk* transcripts are not, however, the only mRNAs that could be subject to *orb* regulation during oogenesis. Several recent studies have identified many other mRNAs that are Orb associated *in vivo* [47, 48]. Included in this group of potential *orb* regulatory targets are mRNAs encoding the Par proteins, aPKC, Baz, Par-6 and Cdc42. Moreover, there is evidence connecting the other fly CPEB protein, Orb2, to the functioning of one of the Par family proteins, aPKC, in cell polarization in the embryonic CNS, in testes and in tissue culture cells [49–51]. These observations prompted us to ask whether *orb* impacts the process of repolarization of the oocyte during mid-stages of oogenesis, and conversely whether the Par proteins, and in particular, aPKC, have any effect on *orb* activity.

## Results

### *orb* hypomorphic allelic combinations display defects in *oskar* mRNA localization

**Fig 1:**
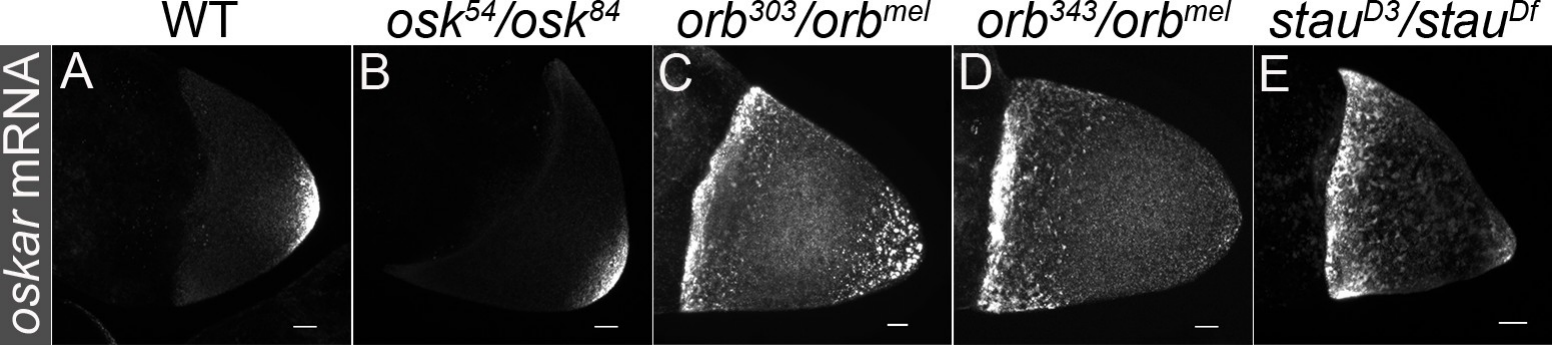
*oskar* mRNA is enriched at the oocyte anterior in *orb* mutants. (A-B) *osk* mRNA localizes to the oocyte posterior in *WT* and *osk* protein null (*54/84*) backgrounds. (C-E) In *orb*^*303/mel*^, *orb*^*343/mel*^ and *stau*^*D3/Df*^ *osk* mRNA is enriched at the oocyte anterior. Images are maximum intensity projections and all scale bars are 10 microns.

In wild type, *osk* mRNA is localized in a tight crescent at the posterior pole of the oocyte after repolarization (Fig 1A) [11, 52]. While *osk* mRNA localization to the posterior is independent of Osk, Osk protein is required to ensure that *osk* mRNA is properly anchored to the posterior cortex [52]. In *osk* protein null mutants, *osk* mRNA is localized at the posterior, but localization is not properly maintained (Fig 1B). While *orb* is required for *osk* mRNA translation, it also plays a role in the proper localization of *osk* message [43, 44]. As shown in Fig 1C, in *orb*^*mel*^/*orb*^*303*^ chambers, the tight localization of *osk* mRNA at the posterior pole is lost. Instead, *osk* mRNA puncta are distributed in a halo around the posterior pole while there is a diffuse pattern of mRNA along the anterior margin of the oocyte. As previously reported, even more extreme defects in *osk* mRNA localization are evident when *orb*^*mel*^ is combined with the null allele *orb*^*343*^ (Fig 1D) [42, 43]. In this allelic combination there is little if any *osk* mRNA at the posterior.

### *orb* does not function in *osk* mRNA transport

**Fig 2:**
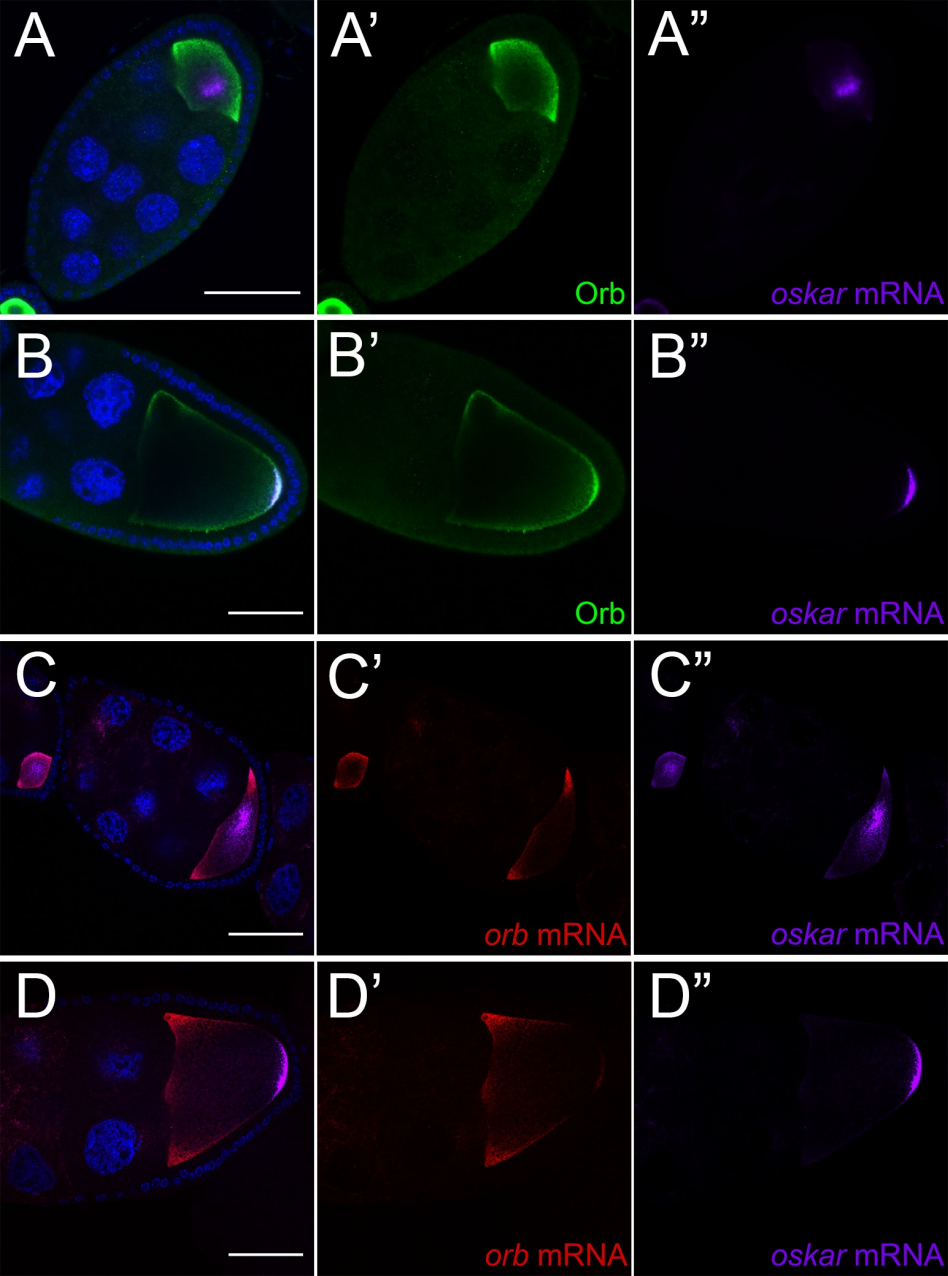
Orb protein and RNA do not colocalize with *oskar* mRNA during transport in wild type oocytes. (A) *osk* mRNA and Orb protein do not co-localize at stage 7. Orb protein is enriched subcortically (A’), while *osk* mRNA is enriched in the middle of the oocyte during transport (A”). (B) *osk* mRNA and Orb protein colocalize at the oocyte posterior at stage 9. (C) *orb* mRNA does not co-localize with *oskar* mRNA at stage 7. *orb* mRNA is enriched at the oocyte anterior (B’) while *osk* mRNA is enriched in the middle of the oocyte (B”). (D) *orb* mRNA does not co-localize with *oskar* mRNA at the posterior at stage 9. Instead, *orb* mRNA is localized along the oocyte anterior (D’), while *osk* mRNA is enriched at the posterior (D”). All scale bars 50 microns.

The *osk* mRNA localization defects in the hypomorphic *orb* mutant combinations resemble those in *staufen* mutants. *staufen* encodes an RNA-binding protein that co-localizes with *osk* mRNA throughout oogenesis, and in *staufen*^*D3/Df*^, *osk* mRNA is partially localized to the posterior and also accumulates at the anterior (Fig 1E) [11, 52, 53]. Thus, one explanation for the defects in *osk* mRNA localization during mid-oogenesis is that *orb* is also required to transport *osk* mRNA [53]. To test this possibility we compared the distribution of Orb protein with that of *osk* mRNA. Prior to stage 7, both *osk* mRNA and Orb protein are localized at the posterior. When the MT network commences repolarization during stage 7, *osk* mRNA transiently accumulates in a cloud near the middle of the oocyte (Fig 2A) [54]. If Orb is directly involved in *osk* mRNA transport, it would be expected to co-localize with *osk* mRNA in this cloud. However, it does not. Instead, most of the Orb is concentrated in the sub-cortical region at the posterior end of the oocyte and along the lateral margins of the oocyte (Fig 2A). Only later, after *osk* mRNA is re-localized to the posterior pole (and presumably being translated) does it again overlap with the posterior cap of Orb protein (Fig 2B). Another *orb* regulatory target is *orb* mRNA and its pattern of localization differs from that of *osk* [55]. In stage 7 chambers, when *osk* mRNA is in the center of the oocyte, *orb* mRNA has a circumferential subcortical distribution around the anterior of the oocyte (Fig 2C). This distribution is maintained at later stages (Fig 2D).

Another indication that *orb* is not directly involved in *osk* mRNA transport comes from the effects of *grk* mutations. In *grk*^*2B/2E12*^ ovaries, posterior follicle cell (PFC) specification is defective and the oocyte fails to initiate repolarization at stage 7 [7, 8]. As a consequence, *osk* mRNA (S1 Fig), the transport protein Staufen, and MT plus ends become enriched in the center of the oocyte, while *bicoid* mRNA localizes to both the anterior and posterior of the oocyte [7, 8]. In this *grk* mutant combination Orb protein and also *orb* mRNA accumulate around the circumference of the oocyte, far from *osk* mRNA (S1 Fig).

### *orb* functions in organizing the oocyte cytoskeleton

An alternative explanation for the mislocalization of *osk* mRNA in *orb*^*mel*^/*orb*^*303*^ and *orb*^*mel*^/*orb*^*343*^ egg chambers is that the cytoskeleton is not properly reorganized during repolarization in the absence of normal *orb* function. This possibility was suggested by the studies of Martin *et al*. ([56]), who showed that in hypomorphic *orb* mutant alleles the oocyte MT network is disrupted and there is premature oocyte cytoplasmic streaming. Several approaches were used to confirm and extend their findings.

#### a) Kinesin-β–galactosidase is mislocalized in orb mutant chambers

**Fig 3:**
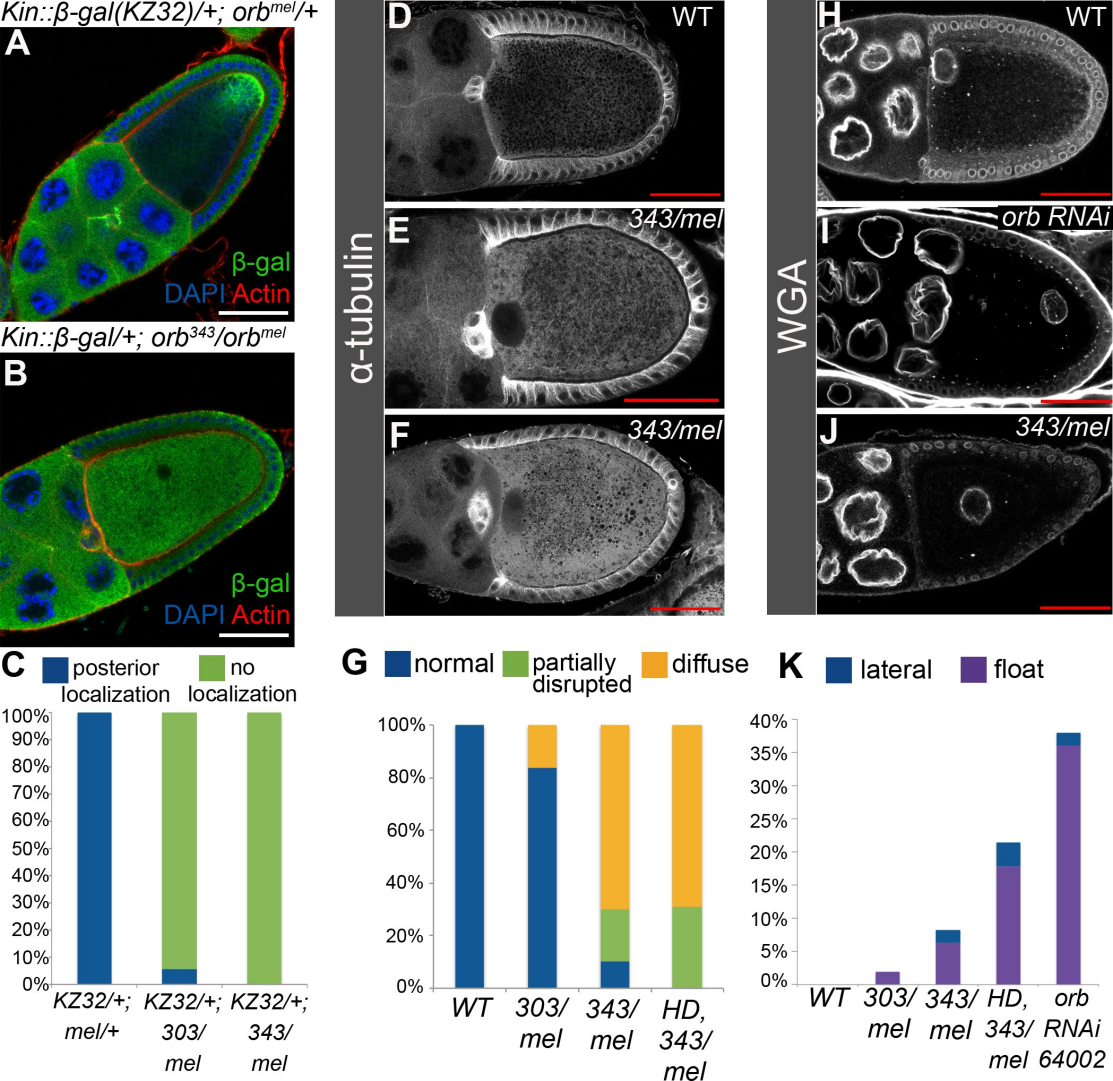
*orb* mutants have defects in microtubule organization and in positioning the oocyte nucleus. (A) Kinesin-βgal (encoded by *KZ32*) is enriched at the posterior of a stage 10 oocyte in an *orb*^mel^ heterozygous background. (B) Kin-βgal does not localize to the posterior at stage 10 in *orb*^*343/mel*^. (C) Frequency of posterior enrichment of Kin-βgal in *orb*^mel^ heterozygotes (n = 52), *orb*^*303/mel*^ (n = 35) and *orb*^*343/mel*^ (n = 41). (D) α-tubulin is enriched in subcortical bundles in a wild type stage 10 oocyte. (E) A *343/mel* stage 10 oocyte in which the α-tubulin bundles are partially displaced from the cortex, and considerable faction of the α-tubulin is distributed throughout the oocyte. (F) A *343/mel* stage 10 oocyte in which α-tubulin is diffuse throughout the oocyte. (G) Quantification of defects in subcortical α-tubulin organization at stages 9–10 wild type (n = 17), *303/mel* (n = 37), *343/mel* (n = 20) and *HD*,*343/mel* (n = 29) oocytes. (H) Wheat germ agglutinin (WGA) staining of wild type shows that the oocyte nucleus is positioned at the dorsal anterior corner. (I) In *orb RNAi (64002)* driven by *maternal* α*-tubulin Gal4 (7062)* the oocyte nucleus is not correctly positioned. (J) *343/mel* also shows defects in the position of the oocyte nucleus. (K) Quantification of defects in oocyte nucleus position at stages 10–11 in wild type (n = 60), *303/mel* (n = 102), *343/mel* (n = 110), *HD*,*343/mel* (n = 84) and *orb RNAi (64002)* driven by *α-tubulin Gal4 (7062)*(n = 50). All scale bars in Fig 3 are 50 microns.

We used the plus-end directed motor Kinesin tagged with **β–**galactosidase (KZ32) to examine the polarity of the MT network [57]. In control oocytes, Kin::β–gal accumulates with the MT plus-ends at the oocyte posterior in a pattern that resembles *osk* mRNA (Fig 3A–3C; S2A Fig) [58]. A quite different result is observed in *orb*^*mel*^*/orb*^*303*^ and *orb*^*mel*^/*orb*^*343*^ egg chambers (Fig 3B & 3C; S2B Fig) [56]. Instead of being enriched at the posterior, Kinesin-**β–**galactosidase is distributed diffusely throughout the oocyte cytoplasm.

#### b) MT network is disrupted in orb mutant chambers

During repolarization, α-tubulin assembles into a network that is in tight association with the anterior and lateral cortical actin (Fig 3D) [20, 59, 60]. Martin *et al*. ([56]) found that this network was partially disrupted in *orb* mutants. To better understand the role of *orb* in organizing the MT network, we examined different allelic combinations. We found that the frequency and severity of the disruption correlates with the extent of reduction in *orb* activity. The least severe effects are observed in *orb*^*mel*^/*orb*^*303*^ egg chambers (Fig 3G). In this genetic background, the MT network is dissociated from the cortex in only about 15% of stages 9–10 oocytes. Similar disruptions in the cortical association of tubulin have been reported for mutants in the actin nucleators *cappuccino* (*capu*) and *spire* (*spir*) [61–64]. The frequency of affected oocytes increases to about 70% when *orb*^*mel*^ is combined with the null allele *orb*^*343*^ (Fig 3E–3G; S2D Fig). In most of the *orb*^*mel*^*/orb*^*343*^ chambers, MTs are diffusely distributed throughout much of the oocyte with little evidence of cortical association (Fig 3E and 3F and S2D Fig). Finally, we combined *orb*^*mel*^ with *orb*^*343*^ and the dominant negative transgene, *HD19*. The *HD19* transgene expresses a chimeric *lacZ*-*orb* 3’UTR mRNA that competes with the endogenous *orb* mRNA [41, 55]. This competition interferes with a positive autoregulatory loop in which *orb* activates its own expression [55]. In *orb*^*mel*^ /*orb*^*343*^ *HD19* ovaries, the MT network phenotypes are fully penetrant (Fig 3G).

As reported by Martin *et al*. ([56]), we found that the defects in the cortical association of the MT network are accompanied by premature cytoplasmic streaming. Examples of normal cytoplasmic movements in a stage 9 egg chamber and oocytes undergoing premature streaming when compromised for *orb* function are shown in supplemental movies (S1–S3 Movies).

#### c) The oocyte nucleus is mispositioned in *orb* mutant oocytes

As the MT network repolarizes in wild type egg chambers, the oocyte nucleus migrates from the posterior of the oocyte to the dorsal anterior corner (Fig 3H; S2E Fig) [7, 8, 22, 65, 66]. Like the MT network, the association of the oocyte nucleus with the dorsal anterior corner is sensitive to reductions in *orb* activity. In the weakest allelic combination, *orb*^*mel*^/*orb*^*303*^, mispositioned oocyte nuclei are observed in less than 5% of stage 9–11 oocytes (Fig 3K). When *orb*^*mel*^ is combined with the null allele, *orb*^*343*^, about 7% of stage 9–11 oocytes have mispositioned nuclei (Fig 3J and 3K; S2F Fig). The frequency of nuclear localization defects increases when *orb* activity is further compromised (Fig 3K). In *orb*^*mel*^*/orb*^*343*^ *HD19* the oocyte nucleus is incorrectly positioned in over 20% of stage 9–11 egg chambers, while it increases to nearly 40% when Orb is depleted by RNAi (#64002) knockdown (S3 Fig) using a driver (maternal α-tubulin Gal4 #7062) that is active during mid-oogenesis (Fig 3I and 3K) [67].

### Are the defects in repolarization due to a failure in *grk* signaling to posterior follicle cells?

Repolarization of the oocyte during stage 7 is triggered by signals emanating from the somatic posterior follicle cells (PFCs). The production of the repolarization signal depends upon the proper specification of the PFCs and this process is orchestrated by the expression of the Grk ligand at the oocyte posterior [7, 8]. Since *grk* mRNA is a known *orb* regulatory target, one explanation for the repolarization defects is that the PFCs are not properly specified when *orb* activity is compromised. To test this possibility we examined the expression of an EGFR dependent enhancer trap, *kekkon-lacZ*, that is activated in follicle cells by *grk* signaling [68–72]. As illustrated for two stage 7 egg chambers in S4A and S4B Fig we found that *kekkon-lacZ* expression in PFCs in *orb*^*mel*^/*orb*^*343*^ egg chambers resembles that in control egg chambers. This result confirms previous studies which showed that anterior follicle cell fate is not duplicated in *orb*^*343/mel*^ egg chambers [7]. While *kekkon-lacZ* expression is unaffected in *orb*^*mel*^/*orb*^*343*^ prior to repolarization, abnormalities are evident at later stages. As shown for a stage 10 *orb*^*mel*^/*orb*^*343*^ chamber in S4C and S4D Fig, expression of *kekkon-lacZ* in dorsal follicle cells is severely reduced compared to the control. This is expected since *grk* signaling to the dorsal follicle cells is known to be disrupted in *orb*^*mel*^/*orb*^*343*^ ovaries [42, 45, 46].

Other observations are also consistent with the idea that the defects in MT organization in *orb* are downstream of both the *grk* dependent specification of PFCs and of the subsequent repolarization signal from the PFCs to the oocyte. For example, in *grk* mutants, *bicoid* mRNA is localized not only along the anterior-lateral margin, but also at the posterior pole[7, 8]. In contrast, when *orb* activity is compromised, localization of *bcd* mRNAs to the posterior pole is not observed (S5 Fig) [42]. The reason for this difference is that in *grk* mutants the PFCs fail to signal the disassembly of the MTOC at the posterior of the oocyte, whereas the posterior MTOC is disassembled in *orb* mutants.

### Genetic interactions between *orb* and the anterior Par genes *aPKC* and *cdc42* disrupt *grk* signaling to dorsal follicle cells

**Fig 4:**
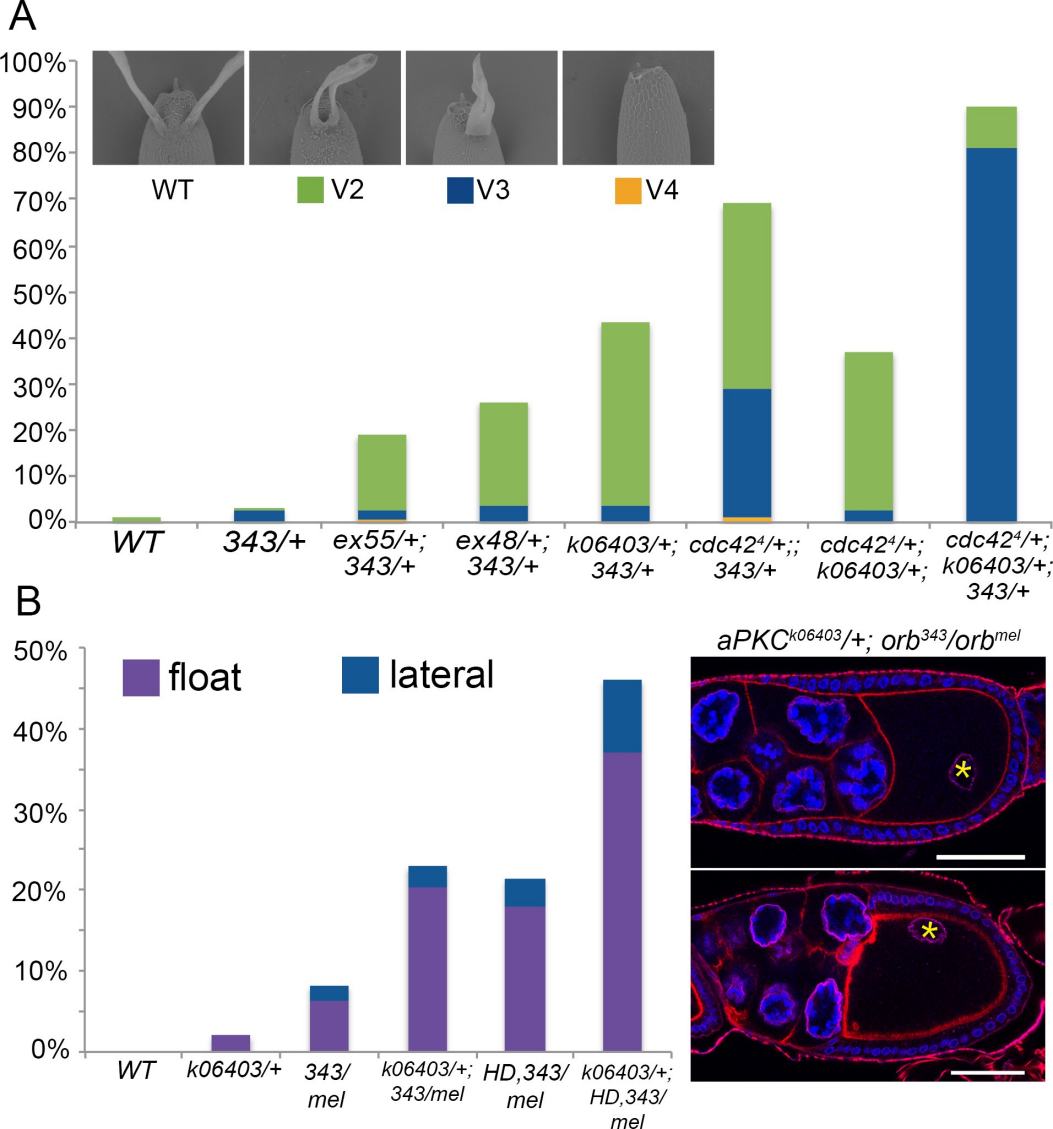
*orb* genetically interacts with *aPKC* for DV polarity and oocyte nucleus position. (A) Top: Dorsal appendage morphology: wild type and ventralized classes V2-V4 [73]. V2 is a reduction in the space between dorsal appendages, V3 is a fusion of the dorsal appendages and V4 is the most severe reduction in dorsal appendages. Bottom: Graph shows the percentage of ventralized eggs laid by wild type, *orb* heterozygotes (*343/+)*, double-heterozygotes between *orb*, *aPKC* alleles (*ex48*, *ex55* and *k06403*), and *cdc42*^*4*^, double-heterozygotes between *cdc42*^*4*^ and *aPKC*^*k06403*^ and triple heterozygotes of *orb*^*343*^, *aPKC*^*k06403*^ and *cdc42*^*4*^. Frequency of DV polarity defects for heterozygous controls not shown in the graph are listed in S1 Table. n = >400 all genotypes. (B) Top: asterisks show mispositioned oocyte nuclei in *aPKC*^*k06403*^*/+; orb*^*343/mel*^ (left shows a “float” position and right shows a lateral position). Scale bars 50 microns. Bottom: *aPKC*^*k06403*^ enhances *orb* mutant defects in oocyte nucleus position. Defects in *aPKC*^*k06403*^*/+* (2%, n = 50), *aPKC*^*k06403*^*/+; orb*^*343/mel*^ (27%, n = 118) and *aPKC*^*k06403*^*/+; orb*^*mel*^*/HD*, *orb*^*343*^ (50%, n = 100). Scale bars are 50 microns.

One explanation for the failure to repolarize the MT cytoskeleton is that *orb* activity impacts either directly or indirectly the functioning of the Par proteins. In fact, precedence for an *orb-*Par connection comes from experiments showing that one of the targets for the other fly CPEB protein, *orb2*, in spermatid cyst polarization and in asymmetric cell division in the embryo is the message encoding the apical Par protein *aPKC* [49, 50]. To explore this idea further we took advantage of the fact *orb* is weakly haploinsufficient for D-V polarity [55]. About 5% of the eggs laid by *orb*^*343*^/+ are ventralized due to defects in translating *grk* mRNA at the dorsal anterior corner of the oocyte (Fig 4A). The frequency of D-V polarity defects can be enhanced by reducing the activity of other genes that are important for *orb* function in *grk* signaling.

We used three different *aPKC* mutants, a strong allele, *k06403*, and two hypomorphic alleles, *ex48* and *ex55*, to test for dominant genetic interactions with *orb* [74, 75]. While the frequency of D-V polarity defects in eggs produced by mothers heterozygous for these three *aPKC* alleles is similar to WT (S1 Table), these mutations substantially enhanced the frequency of D-V polarity defects when *trans* to *orb*^*343*^/+. The weak hypomorphic alleles increase the frequency of ventralized eggs four to five fold (20% and 25%), while the frequency is increased nearly nine fold (44%) by the null allele (Fig 4A).

To extend this analysis, we also asked whether there are genetic interactions between *orb*^*343*^ and the *cdc42* gene, which, like *aPKC*, plays an important role in establishing apical cell polarity [26, 76]. In *orb*^*343*^*/ cdc42*^*1*^ *trans*-heterozygotes there was modest increase (three-fold) in the frequency of D-V polarity defects (S1 Table), while in *orb*^*343*^*/cdc42*^*4*^ *trans-*heterozygotes the frequency of D-V polarity defects increased by nearly fifteen fold (Fig 4A). Consistent with the idea that the effects on *grk* signaling are related, at least indirectly, to the functioning of the Par proteins in the process of repolarization, we also observed genetic interactions between *aPKC*^*k06403*^ and *cdc42*^*4*^. Whereas background levels (~1%) of D-V polarity defects are evident in eggs produced by either *aPKC*^*k06403*^ and *cdc42*^*4*^ heterozygotes, over 35% of the eggs laid by *trans-*heterozygous mothers had D-V polarity defects (Fig 4A).

We also examined eggs produced by females triply heterozygous for *orb*^*343*^, *aPKC*^*k06403*^ and *cdc42*^*4*^. In this triple heterozygote about 90% of the eggs have D-V polarity defects (Fig 4A). As would be predicted, accumulation of Grk protein at the dorsal anterior corner of the oocyte is clearly reduced (S6A–S6C Fig). Interestingly, the oogenesis defects are not restricted to *grk* translation. S6D–S6F Fig also shows that the localization of *osk* mRNA at the posterior pole is also reduced compared to control egg chambers.

### Genetic interactions between *orb* and *aPKC* interfere with positioning of the oocyte nucleus

Further evidence that *orb* might work in conjunction with *aPKC* in the process of repolarization comes from analysis of oocyte nucleus positioning in backgrounds simultaneously compromised for both genes. As described above, oocyte nucleus mispositioning is observed in ~7% of the *orb*^*mel*^/*orb*^*343*^ egg chambers. The frequency of a mispositioned oocyte nucleus increases to nearly 25% of the chambers when *orb*^*mel*^/*orb*^*343*^ females are also heterozygous for *aPKC* ^*k06403*^ (Fig 4B). A similar enhancement is observed when *aPKC* ^*k06403*^ is combined with *orb*^*mel*^/*orb*^*343*^ *HD19G*. In *orb*^*mel*^/*orb*^*343*^ *HD19G* chambers about 20% have a mispositioned oocyte nucleus, while the frequency of oocytes with a mispositioned nucleus increases to nearly 50% when the *orb*^*mel*^/*orb*^*343*^ *HD19G* females are also heterozygous for *aPKC* ^*k06403*^ (Fig 4B). Importantly, *aPKC* on its own is not haploinsufficient for proper oocyte nucleus migration (Fig 4B).

### Localization of *aPKC* mRNA within the oocyte depends upon *orb*

**Fig 5:**
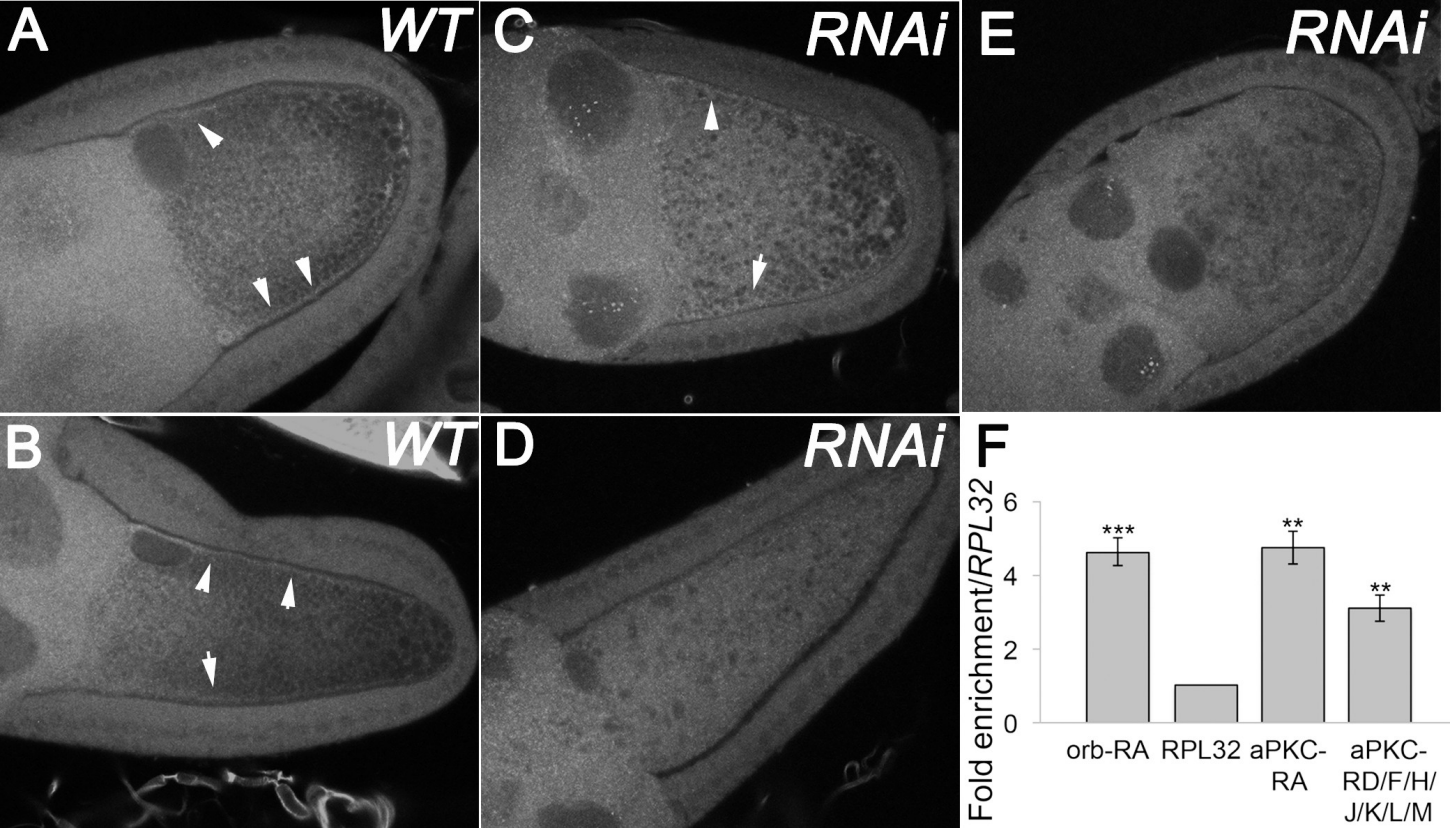
*aPKC* mRNA localization depends on *orb* and Orb binds to aPKC 3’UTRs. (A and B) In wild type egg chambers *aPKC* mRNA is localized along the anterior lateral cortex of the oocyte (arrowheads). (B) When *orb RNAi* (*64002*) is driven by a midstage driver, *maternal α-tubulin Gal4 (7062)*, *aPKC* mRNA localization is patchy along the oocyte cortex (arrowheads), or is not localized along the cortex (D and E). (F) Orb immunoprecipitation followed by RT-qPCR shows enrichment of *orb-RA* 3’UTR and multiple species of *aPKC* 3’UTRs. The enrichment is calculated using ΔΔCT, and *orb* and *aPKC* 3’UTRs are significantly enriched compared to the control 3’UTR, *RPL32* (n = 3 biological replicates).

**Fig 6:**
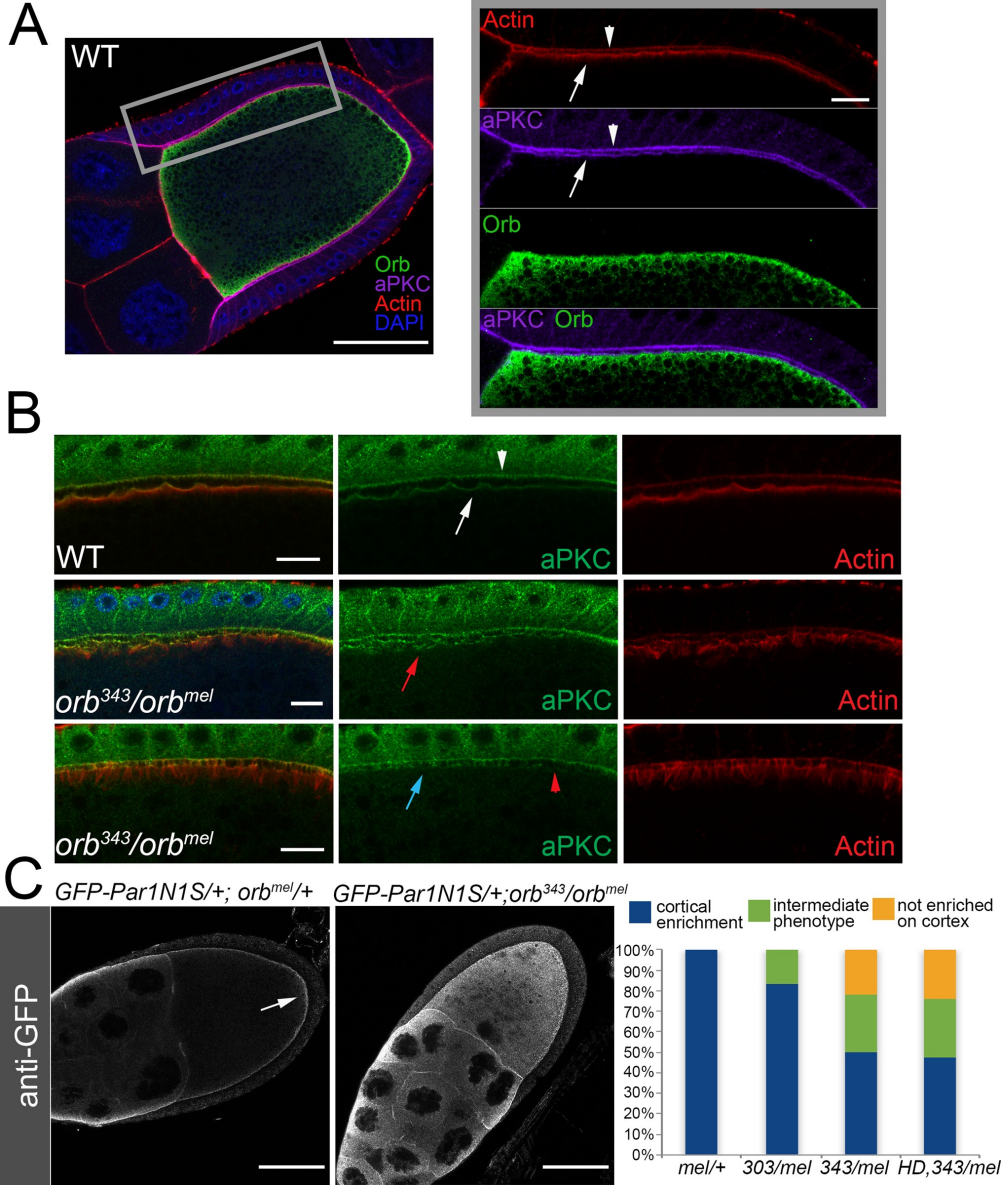
aPKC and Par-1 proteins are not correctly localized in *orb* mutants. (A) Left: A wild type stage 10 egg chamber stained with Orb (green), aPKC (magenta), Actin (red) and DAPI (blue). Scale bar 50 microns. Right: Zooming in on the lateral cortex demonstrates that Actin and aPKC co-localize at the apical domain of the follicle cells (arrowhead) and along the lateral cortex of the oocyte (arrow). Orb localization is subcortical and does not directly co-localize with aPKC or Actin on the cortex. Scale bar 10 mirons. (B) Top: In wild type aPKC protein is observed on the apical side of the follicle cells (arrowhead) and on the anterior lateral cortex of the oocyte (arrow) and colocalizes with actin in both tissues. Middle: In *343/mel* the localization of aPKC and Actin along the anterior lateral cortex of the oocyte is irregular (red arrow). Bottom: Another example of aPKC and Actin localization in *343/mel*. aPKC association is irregular (blue arrow) or diffuse (blue arrowhead). Correlated with the disruptions in the cortical association of aPKC, the actin filament organization in the cortex is abnormal. Instead of the tight actin network at the cortex, there are filaments extending out into the oocyte cytoplasm as well as regions that lack cortical actin Scale bars 10 microns. (C) GFP antibody staining for a GFP-Par-1-N1S fusion protein expressed under the control of a maternal tubulin promoter. GFP-Par-1 localizes to the oocyte cortex in wild type (not shown) and *orb*^*mel*^*/+* (n = 45) egg chambers and is enriched at the posterior (arrow). In contrast, GFP-Par-1 is displaced from the cortex in *343/mel* egg chambers. The graph shows the frequency of defects in GFP-Par-1 localization in *orb*^*mel*^*/+* (0%, n = 45), *orb*^*303/mel*^ (16%, n = 30), *orb*^*343/mel*^ (50%, n = 60) and *orb*^*mel*^*/HD19*, *orb*^*343*^ (53%, n = 38). The intermediate phenotype as described in the graph can be seen in S8 Fig, where there is some patchy cortical enrichment of GFP, and lower levels throughout the oocyte cytoplasm. Scale bars 50 microns.

One plausible explanation for the genetic interactions is that one of the *orb* functions in repolarization is to regulate *aPKC* mRNA. To explore this possibility, we examined the effects of compromising *orb* on the pattern of accumulation of *aPKC* mRNA. While *aPKC* mRNA is present in both somatic and germline cells in wild type ovaries, the highest concentrations of mRNA in the germarium and in stage 1–7 egg chambers are found in the oocyte (S7A and S7B Fig). In stage 9 and older chambers, *aPKC* mRNA is no longer enriched in the oocyte relative to levels in the nurse cells; however, within the oocyte a fraction of the mRNA localizes along the oocyte cortex with the highest levels of *aPKC* mRNA towards the anterior of the oocyte and lower levels towards the posterior (arrows in Fig 5A and 5B and S7C Fig). Orb protein also localizes along the lateral cortex of the oocyte in wild type egg chambers (see Figs 2 and 6), while in chambers compromised for *orb*, Orb association with the cortex is substantially reduced (S3 Fig). Consistent with a role for Orb in anchoring *aPKC* mRNA during mid-oogenesis, we find that the anterior and lateral cortex associated *aPKC* mRNA is either partially (Fig 5C) or largely (Fig 5D & 5E) lost when *orb* activity is depleted by *RNAi*.

### Orb binds to *aPKC* mRNAs

As noted in the introduction, *aPKC* mRNA is one of several thousand mRNAs that are associated with ectopically expressed Orb2 and Orb in tissue culture cells [48]. To determine if *aPKC* mRNA is bound by Orb in ovary extracts, we used immunoprecipitation to isolate Orb associated RNAs. After reverse transcription using an oligo dT primer, we used quantitative PCR to assay for specific mRNA species. For the positive control, we used primers for *orb-RA* 3’UTR which contains four canonical cytoplasmic polyadenylation elements (CPEs: UUUUAU or UUUUAAU). Previous studies have shown that Orb binds to the *orb* mRNA 3’UTR and positively autoregulates its own expression [55]. There are twelve predicted *aPKC* mRNA species with six different predicted 3’UTRs. Four of the six predicted 3’UTRs have canonical CPE sequences. One of these, *aPKC-RA*, has a 3’UTR with three canonical CPEs while the remaining *aPKC* mRNAs (*RD*, *RF RJ*, *RK*, *RL* and *RM*) have overlapping UTRs with 2 canonical CPEs. Fig 5F shows that in ovary extracts both types of *aPKC* 3’UTRs are enriched in Orb immunoprecipitates.

### Distribution of aPKC along the anterior lateral cortex depends upon *orb*

We next examined the pattern of accumulation of aPKC protein. In wild type stage 10–11 oocytes, aPKC protein is localized to the anterior-lateral cortex where it appears to be in close association with the cortical actin network (Fig 6A) [37]. Except for this cortically localized protein, there is little aPKC elsewhere in the oocyte. Orb is localized just interior to the cortical actin-aPKC layer (Fig 6A). aPKC is also localized along the apical surface of the somatic follicle cells facing the germline, and in confocal images the somatic and oocyte aPKC proteins typically appear as a set of parallel tracks along the anterior-lateral cortex (Fig 6A).

The pattern of aPKC localization in the oocyte is altered when *orb* activity is compromised. Instead of a tightly organized track coincident with cortical actin, aPKC protein distribution becomes irregular and patchy (Fig 6B). In some regions, there are small gaps (Fig 6B: lower panel: blue arrowhead) while in other regions aPKC is missing altogether (Fig 6B, lower panel: red arrowhead). In other cases, the aPKC protein extends from the cortex into the interior of the oocyte (Fig 6B, middle panel: red arrowhead).

### Par-1 protein localization is also disrupted

The effects of reducing *orb* activity on aPKC localization within the oocyte, taken together with the genetic interactions between *orb*, *aPKC* and *cdc42* indicate that *orb* is required for the proper functioning of anterior Par proteins. It seemed possible that the posterior Par proteins might also be dependent on *orb*. To test this idea, we examined the localization of a Par-1-GFP fusion protein that is expressed in the germline. In control stage 8–11 oocytes, the Par-1-GFP fusion protein localizes along the oocyte cortex and tends to be enriched towards the posterior of the oocyte. Fig 6C and S8 Fig show that like aPKC, Par-1-GFP localization depends upon *orb*, and is disrupted when *orb* activity is compromised. The extent of disruption is correlated with the severity of the reduction in *orb* activity. In *orb*^*mel*^*/orb*^*303*^, a small percentage of the chambers have an obvious, but not complete loss of Par-1-GFP association with the oocyte cortex (Fig 6C). Even more extensive alterations are observed in *orb*^*mel*^*/orb*^*343*^ and *orb*^*mel*^*/orb*^*343*^ *HD19G* chambers. In these genetic backgrounds, more than half of the egg chambers show either a reduction (S8B Fig) or complete loss of cortical Par1-GFP (Fig 6C and S8C Fig).

### Cortical Actin cytoskeletal organization requires *orb*, *aPKC* and *cdc42*

In addition to its functions in Par dependent polarity, the apical Par protein Cdc42 can also activate effectors of the actin cytoskeleton (Cip4, WASp and Arp23). Studies by Leibfried *et al*. ([26]) have shown that one of the important Cdc42 targets during oocyte repolarization is the actin cytoskeleton. When *cdc42* activity is compromised, the organization of cortical actin is disrupted. While the apical Par proteins aPKC and Baz are not thought to have a direct role in modeling the actin cytoskeleton, they are required for Cdc42 localization. As a consequence, *aPKC* and *baz* mutants have equivalent defects in the anterior lateral cortical domain. For these reasons, we wondered whether *orb* function might also impact the organization of the cortical actin cytoskeleton during repolarization.

**Fig 7:**
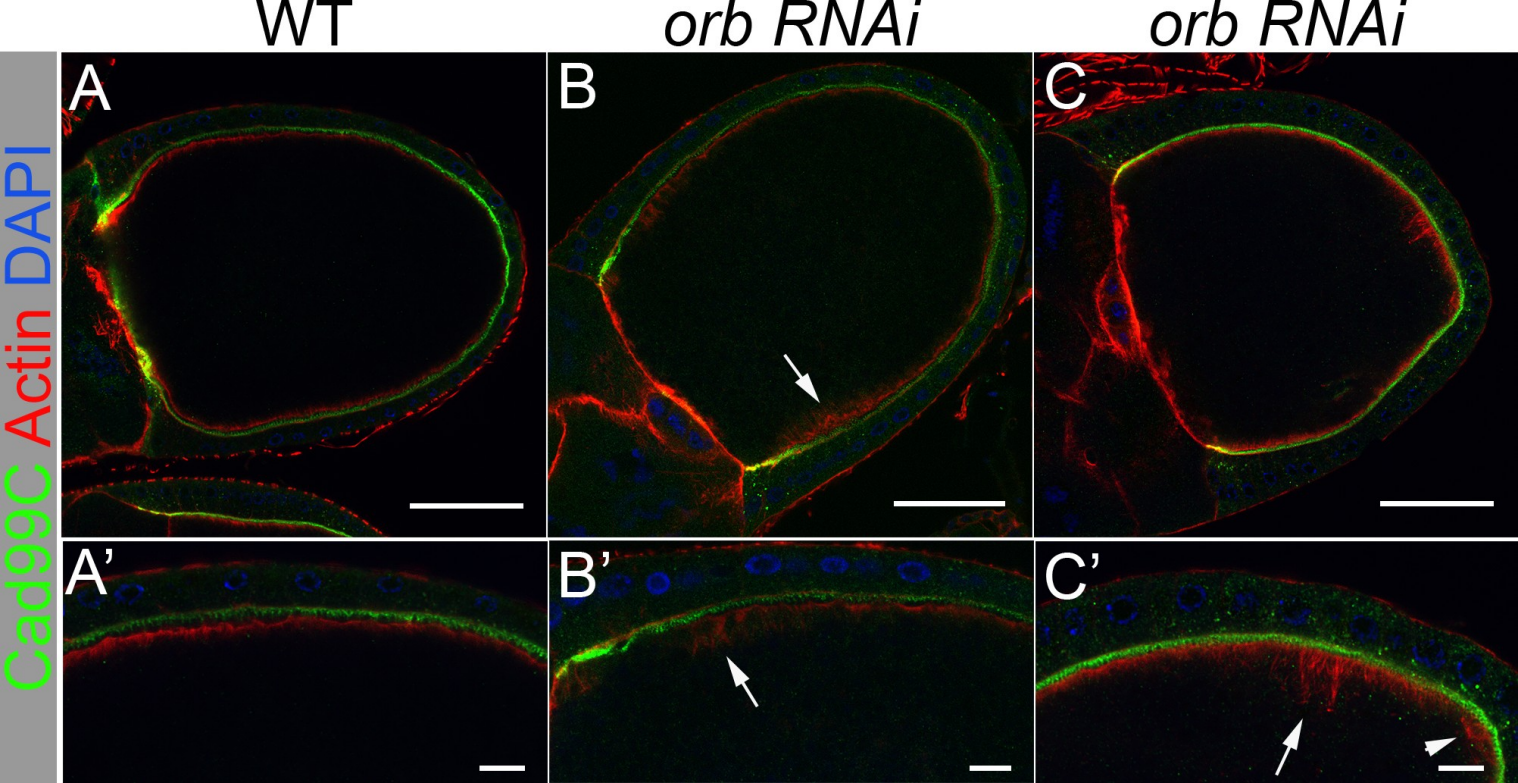
The oocyte actin cytoskeleton is not maintained in *orb*. Egg chambers are stained with Cad99C (green), which labels the apical microvilli of the somatic follicle cells, and Phalloidin (red), which labels Actin on both the apical side of the follicle cells and the oocyte cortex, and DAPI (blue). (A and A’) Wild type organization of Actin along the oocyte cortex (n = 16). (B and B’) Defects in Actin organization in *orb*^*mel*^*/orb*^*343*^ are observed in 72% of oocytes stages 9–11 (n = 18). (C and C’) Defects in actin organization when *orb* RNAi (#64002) is expressed during mid-stages of oogenesis (maternal α-tubulin Gal4, #7062) are observed in 65% of oocytes stages 9–11 (n = 20). We also observed a defect in actin structure at a frequency of 70% in *HD19*, *343/mel* oocytes stages 9–11 (n = 10). Arrows point to defects in cortical actin organization as described in the text. Top panel scale bars are 50 microns; bottom panel scale bars are 10 microns.

To address this question we examined the cortical actin cytoskeleton in *orb*^*mel*^/*orb*^*343*^ and in *orb* RNAi egg chambers. In the experiment in Fig 7, we labeled follicle cells membranes with Cadherin 99C (Cad99C) antibodies, while the actin cytoskeleton was labeled with phalloidin [77]. In wild type, actin is enriched along anterior oocyte margin and the anterior lateral cortex (Fig 7A and 7A’) [26]. The tight association of actin along the oocyte cortex seen in wild type chambers is disrupted when *orb* activity is compromised in either *orb*^*mel*^*/orb*^*343*^ oocytes (Fig 7B and 7B’) or when *orb* RNAi is expressed during midstages by a maternal α-tubulin driver (#7062) (Fig 7C and 7C’). In some regions, the actin matrix is displaced from the cortex (Fig 7B, arrow). In other regions, there are “flares” of actin filaments that extend out from the cortical actin matrix into the ooplasm (Fig 7C’, arrow). The matrix can also unravel forming small bubbles (Fig 7C’, arrowhead) or even disappear completely (Fig 7B’). These defects could be due to a failure to properly crosslink the cortical actin bundles.

aPKC association with the oocyte cortex is thought to depend upon the integrity of the cortical actin cytoskeleton [26]. This raises the possibility that the defects in aPKC localization in *orb* mutants might be connected to abnormalities in the cortical actin cytoskeleton. The results shown in Fig 6B indicate that this is likely to be the case. In regions where the cortical actin matrix is disrupted, aPKC association with the cortex is reduced or lost. There seems to be a similar connection between the severity of the defects in Par-1 localization and the extent of the abnormalities in the cortical actin cytoskeleton (see S8 Fig). The *orb*^*mel*^*/orb*^*343*^ chambers that have most extensive perturbations in the cortical actin cytoskeleton have more pronounced defects in Par-1 localization (S8C Fig) than in chambers in which the cytoskeleton defects are less severe (S8B Fig).

### Patronin and Shot association with the anterior-lateral oocyte cortex is *orb* dependent

**Fig 8:**
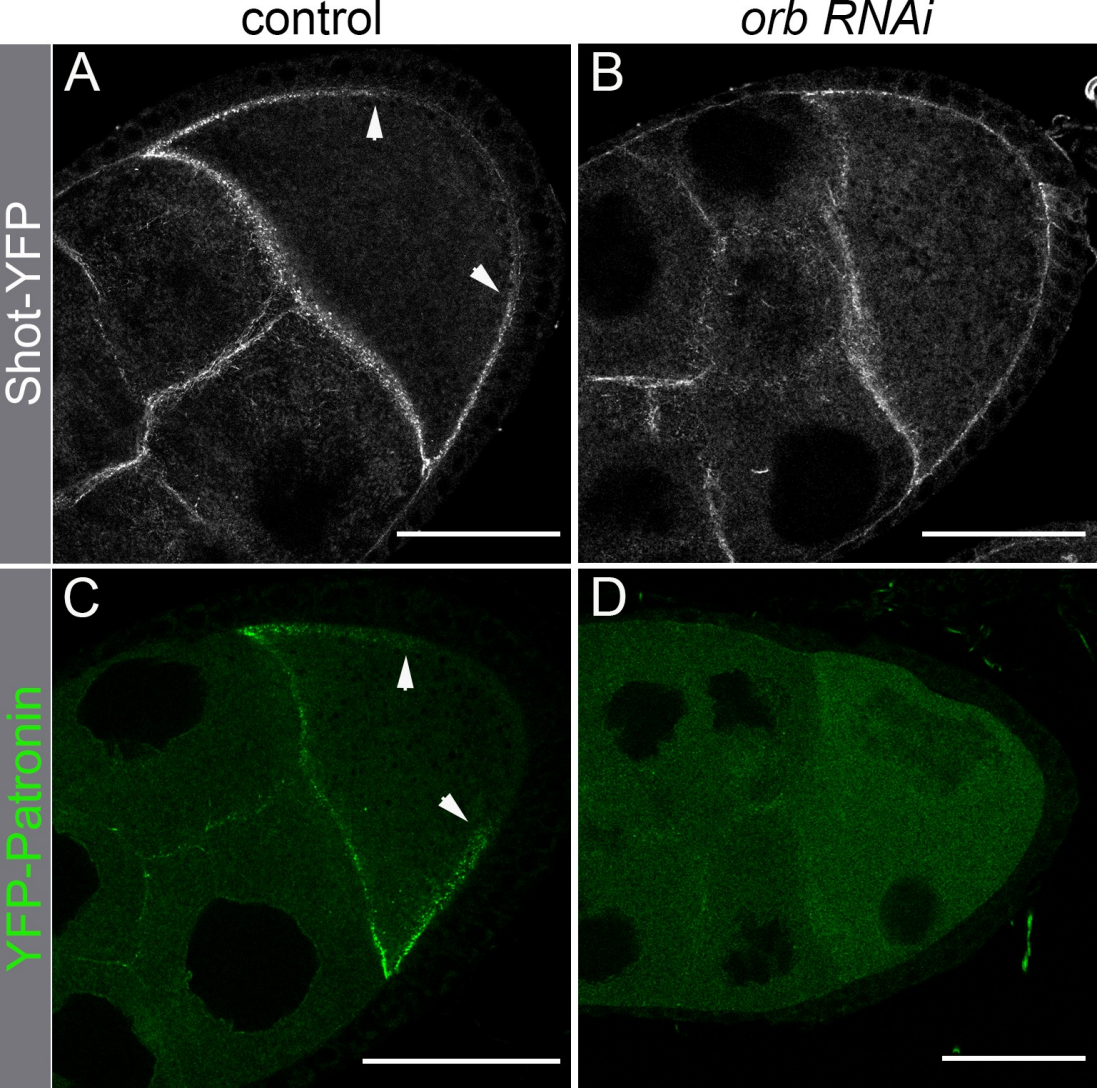
Shot and Patronin cortical association is disrupted when *orb* activity is compromised. *orb* RNAi (*64002*) was expressed during mid-stages of oogenesis using the maternal α-tubulin Gal4 (*7062*) driver in flies carrying a BAC transgene with *shot* tagged with a C-terminal YFP, or a maternally expressed *YFP-patronin* transgene[21]. (A, C) In maternal α-tubulin Gal4 control stage 9 egg chambers, Shot-YFP and YFP-Patronin associate with the anterior and anterior lateral cortical actin network. As indicate by the arrows, only very low levels of these proteins localize to the posterior cortex. (B,D) When *orb* activity is compromised by RNAi knockdown, the association of Shot-YFP and YFP-Patronin with the anterior and anterior-lateral cortex is disrupted. Scale bars are 50 microns.

The repolarization of the MT network during mid-oogenesis depends upon the MT binding protein Patronin and its association with the actin-MT linker Shot. Since the integrity of the cortical actin network is disrupted when *orb* activity is compromised, we wondered whether Patronin and Shot association with the oocyte cortex is also affected. To investigate this possibility, we compared the localization of Shot-YFP expressed from a BAC transgene and YFP-Patronin expressed from a germline specific *mattub* promoter (Fig 8) ([21]) in wild type egg chambers and in chambers in which *orb* activity was knocked down by RNAi.

In wild type stage 9–11 oocytes Shot and Patronin are found associated with the oocyte cortex (Fig 8, S9 and S10 Figs) [21]. In the oocyte, Shot and Patronin are localized in a punctate pattern just underneath the cortical actin network (S9 and S10 Figs). Shot-YFP (S9 Fig) and Patronin-YFP (S10 Fig: expressed as an endogenously tagged protein) also localize to the apical surface of the follicle cells, and these two proteins appear as a parallel track along the lateral surface of the oocyte with the cortical actin network in between. Both Shot-YFP and YFP-Patronin are enriched along the anterior and anterior-lateral cortex, while they are absent from the posterior cortex (Fig 8A–8C; arrowheads). When *orb* activity is knockdown by RNAi, the association of Shot-YFP and YFP-Patronin with the anterior-lateral cortex of the oocyte is disrupted and much of the protein is instead distributed in the ooplasm (Fig 8B and 8D). Similar, though not quite as severe alterations in the cortical association of Shot-YFP and Patronin-YFP are observed in stage 9–11 *orb*^*343*^*/orb*^*mel*^ egg chambers (S9B and S9B’ Fig and S10B–S10B” Fig).

### *orb* and *aPKC* are functionally interdependent

**Fig 9:**
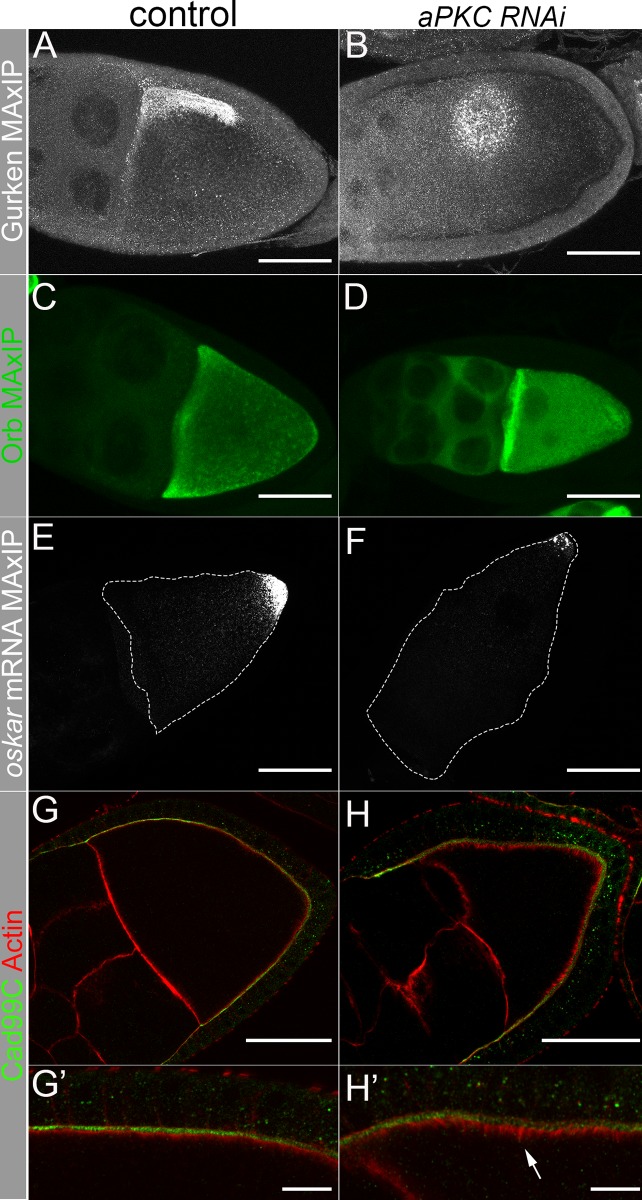
Germline expression of *aPKC* RNAi *(35140)* at midstages of oogenesis leads to germline polarity defects. Maximum intensity projections show that the patterns of Gurken protein(A-B), Orb protein(C-D) *osk* mRNA(E-F), and actin (G-G’,H-H’) are altered when *aPKC RNAi (35140)* is driven by *maternal α-tubulin Gal4* (*7063*). In this background, the oocyte nucleus is not always positioned at the dorsal anterior corner and Gurken protein remains localized with the oocyte nucleus. Orb protein accumulates along the anterior margin of the oocyte and *osk* mRNA is reduced at the oocyte posterior. Like *orb*, the stereotypical organization of the cortical actin network is perturbed. A-H scale bars are 50 microns; G’ and H’ scale bars are 10 microns. (G-G’,H-H’) actin is red and Cad99C is green.

Par proteins establish and maintain polarity within a cell by both positive and negative cross-regulatory interactions. For this reason it seemed possible that *aPKC* and *orb* function in the oocyte might be mutually interdependent. To explore this possibility we used the mid-oogenesis *GAL4* driver maternal α-tubulin (7063) to express *aPKC* RNAi (*35140*). In this background, we observed that the oocyte nucleus is mispositioned in 69% of the stage 9–11 egg chambers when *aPKC* activity is depleted. Accompanying the oocyte nucleus position defects, Gurken protein is mislocalized with the oocyte nucleus (Fig 9A and 9B). Additionally, there are alterations in the pattern of Orb protein accumulation. Instead of being distributed subcortically along the entire surface of the oocyte, high levels of Orb accumulate at the anterior oocyte-nurse cell margin (Fig 9C and 9D). There is also a reduction in the posterior cap of *osk* mRNA compared to wild type (Fig 9E and 9F). Similar effects on the positioning of the oocyte nucleus and the localization of polarity markers (Staufen and Vasa) have been reported for *cdc42* [26]. Moreover, like *orb* and *cdc42*, the cortical actin network is also perturbed in the *aPKC* knockdown (Fig 9H and 9H’).

### aPKC regulates orb

The alterations in the pattern of Orb protein accumulation in the RNAi knockdown experiments prompted us to ask whether *aPKC* impacts *orb* autoregulation. Orb promotes its own expression through sequences in the *orb* mRNA 3’UTR. When the *orb* 3’UTR is linked to coding sequences for *E*. *coli* β-galactosidase in the *HD19* transgene, expression of β-galactosidase becomes dependent upon *orb* activity [55]. S11A Fig shows that β-galactosidase expression from the *HD19* (*hsp83*: *lacZ*-*orb* 3’UTR) transgene is also dependent upon *aPKC* activity. In the *aPKC* mutant combination, *aPKC*^*k06403*^*/aPKC*^*ex48*^, β-galactosidase expression is reduced about two-fold compared to the control (S11B Fig).

Like CPEB proteins in other species, *orb* activity is regulated by phosphorylation [78]. In wild type ovaries, there are multiple phosphorylated isoforms. On standard SDS polyacrylamide gels these different Orb isoforms typically resolve into a closely spaced doublet with the more heavily phosphorylated isoforms migrating more slowly (S11C Fig). In S11C Fig, we compared the relative yield of the upper (more phosphorylated) and lower (less phosphorylated) bands in wild type and *aPKC*^*k06403*^*/aPKC*^*ex48*^ mutant ovaries. In the *aPKC*^*k06403*^*/aPKC*^*ex48*^ the ratio of upper to lower bands is reduced compared to wild type (S11D Fig).

## Discussion

Previous studies have implicated *orb* in the translational regulation of *osk* and *grk* in the stages following the repolarization of the MT network [43–45]. In addition, the proper localization of these mRNAs also depends *orb* activity [42–45]. This observation led to the idea that in addition to controlling translation, *orb* might also have a role in transport and/or anchoring of these mRNAs once they were properly localized. While our results argue against a direct role in transport, they support the idea that the mislocalization of *osk* and *grk* mRNAs when *orb* activity is compromised during mid-oogenesis arises at least in part because *orb* is required for the proper organization of both MTs and the cortical actin cytoskeleton.

The reorganization of the MT network after stage 7 is a multistep process. It begins with a signal from the PFCs that induces the disassembly of the MTOC that is located just posterior to the oocyte nucleus. The production of this somatic signal depends upon the proper specification of the PFCs, and PFC specification requires the expression of Grk protein at the posterior pole of oocyte earlier in oogenesis [7, 8]. Translation of *grk* mRNA at the posterior pole during stages 1–7 depends upon *orb*, and consequently it functions upstream of PFC specification. However, in our experiments *orb* activity prior to stage 7 is not limiting, and sufficient amounts of Grk are expressed to properly specify PFCs [7]. Thus, the defects that we observe in oocyte repolarization when *orb* activity is compromised during mid-oogenesis are downstream of both the *grk* signal to the posterior follicle cells and the signal from the PFCs to the germline that induces MTOC disassembly.

Three other findings are consistent with this conclusion. First, when PFCs are not properly specified, the posterior MTOC fails to disassemble [7, 8, 18]. By contrast, when *orb* activity is compromised during mid-oogenesis the MTOC dissembles as in wild type. Second, the formation of a non-centrosomal cortical based MT network is initiated along the anterior/lateral margin of the oocyte even in the absence of the PFC signal. This is not true in our experiments; the anterior/lateral MT network is not properly established. Third, in the absence of the PFC signal, Staufen protein, Kinesin-β-gal and *osk* mRNA concentrate in the center of the oocyte, while *bcd* mRNA is found not only at the anterior but also at the posterior end of the oocyte. In contrast, in *orb* mutants, *osk* and also *bcd* mRNA accumulate at the anterior of the oocyte, while Kinesin-β-gal is unlocalized.

As the posterior MTOC is disassembled, a MT network emanating from the anterior and anterior-lateral cortex of the oocyte is established. The initiation of this non-centrosomal cortical based MT network is mediated by the spectraplakin, Shot, and the minus-end MT binding protein, Patronin [21]. Shot associates with the actin rich anterior and anterior lateral cortex and recruits Patronin. Patronin then nucleates the assembly of the MT network. Nashchekin *et al*. ([21]) have shown that proper polarization of the MT network by Shot and Patronin depends upon the Par protein Par-1. By an unknown mechanism, Par-1 blocks Shot association with the actin rich cortex. Since Par-1 is enriched around the posterior cortex of the oocyte, this restricts the *de novo* assembly of MTs to the anterior and anterior-lateral cortex. While Par-1 is required to exclude Shot from the posterior cortex, the *de novo* assembly of MTs requires Shot association with the anterior and anterior-lateral cortex. This presumably does not happen when *aPKC*, *cdc42* and/or *baz* are compromised in the germline because the anterior and anterior-lateral cortical actin network is disrupted.

Our results place *orb* upstream of Shot and Patronin and suggest that the defects in oocyte MT repolarization likely arise for several reasons. One would be defects in the localization and functioning of the Par gene products. When *orb* activity is compromised, the association of the Par protein Par-1 with the posterior and aPKC with the anterior-lateral cortex is disrupted. In the absence of proper cortical association, the cross-regulatory interactions between the anterior and posterior Par proteins would be expected to be ineffective. Also consistent with a role for *orb* in the functioning of the Par proteins in MT repolarization are genetic interactions between *orb* and genes encoding the anterior Par proteins, *aPKC* and *cdc42*. *orb* is weakly haploinsufficient for the *grk* signaling pathway, and about 5% of the eggs laid by *orb*^*343*^*/+* females, are ventralized. This weak haploinsufficiency is enhanced when the *orb*^*343*^ mutation is *trans* to mutations in either *aPKC* or *cdc42*. For the *aPKC* null allele, *aPKC* ^*k06403*^, the frequency of ventralized eggs increases to nearly 50%, while about 70% of the eggs laid by females *trans*-heteozygous for *orb*^*343*^ and *cdc42*^*4*^ are ventralized. Moreover, while females heterozygous for either *aPKC* ^*k06403*^ or *cdc42*^*4*^ alone do not lay ventralized eggs, nearly 40% of the eggs laid by females *trans-*heterozygous for these two mutations are ventralized. As we found for *orb*, the localization of the oocyte nucleus to the dorsal anterior corner of the oocyte depends upon *cdc42* and *aPKC*. Leibfried *et al*. ([26]) found that the oocyte nucleus is mispositioned in egg chambers homozygous for *cdc42*^*4*^, while we have shown here that the oocyte nucleus is mispositioned when *aPKC* is knocked down by RNAi. Moreover, the frequency of mispositioned nuclei in *orb*^*mel*^*/orb*^*343*^ is enhanced when the females are also heterozygous for mutations in *aPKC*.

At least some of the effects of *orb* on the Par proteins could be direct. Thus, *aPKC* mRNAs contain CPEs in their 3’UTRs and we have found that *aPKC* mRNA is bound by Orb protein in ovary extracts. Moreover, the distribution of *aPKC* mRNA within the oocyte is altered when *orb* activity is compromised. Interestingly, mRNAs encoding the three other anterior Par proteins, *cdc42*, *baz*, and *par-6* also have CPE motifs in their 3’UTRs and are bound by ectopically expressed Orb in tissue culture cells [48]. Thus, the localization and translation of these Par mRNAs could be regulated by or dependent upon *orb*. In addition, there appears to be a reciprocal relationship between *orb* and anterior Par proteins. This is suggested by the synergistic genetic interactions between *orb* and the Par genes encoding *aPKC* and *cdc42*. It also fits with our finding that *orb* autoregulatory activity and the phosphorylation status of Orb are impacted by *aPKC* depletion.

There are also likely to be indirect effects on the functioning of the Par proteins that in turn perturb the organization of the MT network. For example, Leibfried *et al*. ([26]) have shown that there is a mutually interdependent relationship between the Par proteins and the actin cytoskeleton. They found that Cdc42 localization along the anterior and anterior-lateral cortex of the oocyte depends upon the integrity of the cortical actin network. Conversely, the assembly of the cortical actin network requires *cdc42*, *aPKC* and *baz*. In fact, one of the more striking phenotypes in *orb* mutant oocytes is the disorganization of the cortical actin network. As was observed for *cdc42* [26], the disruptions in the actin network are accompanied by the mislocalization of aPKC. Given the interdependence of the Par proteins and the actin network the disruption of the actin cytoskeleton in *orb* mutants could be due to the misexpression of the Par proteins. However, the Par proteins need not be the only or even the key targets for *orb* regulation of the actin cytoskeleton. For example, the formation of the cortical actin network during mid-oogenesis depends upon two actin nucleators, *capu* and *spir* [61–64]. Mutations in these two genes have a number of phenotypes in common with *orb*. The actin cytoskeleton is fragmented and this in turn leads to a failure to properly organize the MT network and localize *osk* and *grk* mRNAs. Moreover, as has been reported for *orb* [56], premature cytoplasmic streaming is observed in *capu* and *spir* mutant egg chambers. Like the Par proteins, the mRNAs encoding *capu* and *spir* are bound by ectopically expressed Orb in tissue culture cells, and thus could be targets for *orb* regulation. On other the hand, there are some notable differences. In contrast to *orb*, *aPKC* and *cdc42*, *capu* and *spir* eggs are dorsalized not ventralized. Additionally, Par-1 localization to the posterior and lateral cortex does not appear to depend upon *capu* or *spir* [62] whereas it is disrupted in *orb* mutant chambers. Moreover, the effects of *orb* on the actin cytoskeleton need not be limited to these proteins. The mRNA encoding the actin effectors Cip4 and WASp have CPEs in their 3’UTRs and are bound by ectopically expressed Orb in tissue culture cells [48]. Defects in the expression of these proteins would interfere with the remodeling of the anterior/anterior-lateral cortical actin cytoskeleton and consequently disrupt Par dependent MT polarization. Finally, *orb* could also act downstream of the Par proteins. Like *cip4* and *WASp* mRNAs, the mRNAs encoding the MT assembly factors, *shot* and *patronin*, have CPEs in their 3’UTRs and are bound by ectopically expressed Orb in tissue culture cells [48]. Insufficient levels of these factors would be expected to slow or block the *de novo* assembly of MTs along the anterior-lateral cortex.

Thus, a plausible idea is that the defects in the repolarization of the MT network when *orb* is depleted during mid-oogenesis are likely the consequence of the cumulative effects of misregulating mRNAs encoding not only Par proteins but also proteins involved in organizing the actin cytoskeleton and assembling MTs. Because the MT and actin cytoskeleton regulators have interdependent functions, even small perturbations in the abundance of multiple players could lead to wide ranging disruptions in cytoskeletal organization. That mRNA localization/translational regulation might impact the reorganization of the egg chamber after stage 7 at multiple levels is supported by recent studies on *egalitarian* (*egl*). Sanghavi *et al*. ([67]) report that knocking down *egl* just before the MT network in the egg chamber is repolarized induces many of the same phenotypic abnormalities and disruptions in cytoskeletal organization that we have observed when *orb* activity is compromised during mid-oogenesis. Egl together with the Bicaudal-D (BicD) protein loads mRNAs onto a Dynein motors [79–81]. This mRNA cargo complex is responsible for localizing mRNAs in somatic cells and in developing egg chambers. Like Orb, the Egl-BicD cargo complex interacts with many different mRNA species including *orb*. For this reason, loss of *egl* activity is likely to have a global impact on mRNA transport and consequently the localized production of a diverse array of factors needed for the reorganization of the oocyte cytoskeleton during mid-oogenesis.

## Materials and methods

### Drosophila strains

Endogenously tagged Patronin-YFP, YFP-Patronin expressed from a maternal tubulin promoter and Shot-YFP ([21]) were gifts from Daniel St Johnston and Dmitry Nashchekin; *osk*^*54*^, *osk*^*84*^, *stau*^*D3*^, *stau*^*Df*^, *KZ32 (Kinesin-β-gal)* are gifts from Elizabeth Gavis; *grk*^*2B*^, *grk*^*2E12*^, *BB142 (kekkon-lacZ)* are gifts from Trudi Schupbach; *aPKC* mutant alleles *aPKC*^*k06403*^, *aPKC*^*ex55*^, *aPKC*^*ex48*^ and *mattub-GFP-Par-1-N1S* are gifts from Yu-Chiun Wang and Eric Wieschaus; *cdc42*^*1*^ and *cdc42*^*4*^, *aPKC RNAi 35140*, *maternal alphaTubulin67C Gal4 (7062* and *7063*) from Bloomington Stock Center.

### Eggshell phenotype scoring

Eggs were collected by placing flies of the appropriate genotype into cups and were kept at 18 degrees and given fresh apple juice and yeast paste plates daily. The eggshell phenotypes were scored starting on day 3.

### Fluorescence *in situ* hybridization

*osk* FISH probes were a gift from Shawn Little at University of Pennsylvania [54].

*orb* FISH probes were ordered from Biosearch Technologies, and *orb* probes and *aPKC-com* FISH probes (from Xu *et al*. [49]) were coupled to Atto NHS-Ester 565 or 633 (Sigma) and purified using HPLC.

### Immunocytochemistry/antibody staining

Antibodies used were as follows: mouse anti-Orb (4H8, 6H4) used 1:30 each, mouse anti-Gurken (1D12) used 1:20, mouse anti-β-gal (401A) used 1:10, mouse anti-Bic-D (1B11, 4C2) used 1:20 each from the Developmental Studies Hybridoma Bank; rabbit anti-Cadherin99C used 1:1000 was a gift of Dorothea Godt; mouse monoclonal anti-α-tubulin-FITC (clone DM1A) from Sigma; rabbit anti-aPKC (clone c-20, sc-216) used 1:1000 from Santa Cruz Biotechnology. Wheat germ agglutinin (Alexa Fluor 633, Molecular Probes), Phalloidin (Alexa Fluor 546 or 633, Molecular Probes) and DAPI (Molecular Probes) were used.

Secondary antibodies used were goat anti-mouse IgG Alexa 488, 546 or 647, goat anti-rabbit Alexa 488, 546 or 647 (Molecular Probes). Samples were mounted using aqua polymount (Polysciences) on slides and visualized on a Leica SP5 or Nikon A1 confocal microscope.

### Live imaging

Cytoplasmic movements were imaged in live oocytes in halocarbon oil on a Nikon A1 inverted confocal microscope. An image was collected every 5 seconds for at least 2 minutes to visualize cytoplasmic streaming.

### Immunoprecipitation and RNA extraction

Mouse anti-Orb (4H8 and 6H4) or mouse anti- β-gal (401A) were coupled to A/G agarose beads (Santa Cruz Biotechnology) by incubating overnight at 4 degrees. 250 females were dissected in ice cold 1xPBS and ovaries were transferred to dry ice while dissecting. RNAsin (Promega) was added to ovaries and they were crushed using a plastic pestle to make a paste. The ovary paste was centrifuged at 3000 rpm for 5 minutes at 4 degrees twice, and the supernatant was saved. Half of the supernatant was added to the Orb antibody coupled with beads, and the other half was added to the control antibody beads. CoIP buffer ([55]) and RNasin (Promega) was added to IPs, which were left to rotate for 3 hours at 4 degrees. The beads were pelleted by centrifugation and washed with coIP buffer 5 times.

RNA was released from the beads by adding 10 mM HEPES 1% SDS solution and β-mercaptoethanol, and left in a 65 degree water bath for 15 minutes. Phenol followed by phenol chloroform was used for extraction, and the water phase was ethanol precipitated with glycogen added as a carrier. The pellet was dried and then DNAse (Promega) treated.

### RT-qPCR

The RNA samples were incubated with oligodT (IDT) at 65 degrees for 10 minutes. AMV reverse transcriptase (Promega) reactions were set up, and for each IP a control reaction was set up without reverse transcriptase. The samples went through the following program for reverse transcription on a PCR machine: 55 degrees for 1 min, 48 degrees for 30 min, 55 degrees for 15 min, 95 degrees for 5 min, hold at 4. For quantitative PCR, Power CybrGreen PCR master mix (Life Technologies) was used. Each qPCR reaction was done in triplicate and the average CT was used. The control samples without reverse transcriptase were also run to confirm the DNase treatment worked. The amplification of target 3’UTRs from the Orb IP were compared to the amplification from the control IP and normalized to a control (*RPL32*) to calculate ΔΔCT.

### Westerns

For the Western blots to measure levels of β-gal expression ovaries were dissected in PBS and frozen on dry ice. Frozen tissue was crushed with a pestle in SDS buffer with urea, boiled and spun down. The extracts were loaded on a 10% SDS-Page gel. Proteins were transferred to a PVDF membrane and the membrane was cut to blot for β-gal and BEAF. For the phosphorylated Orb isoforms, ovaries from two female flies were dissected in 100 ul of 1X PBS. The ovaries were immediately transferred to 40 ul of 2XSDS buffer (100 mM Tris-Cl; 4% SDS, 200 mM DTT and 0.2% bromphenol blue) and boiled. A second set of ovaries were dissected, transferred to the same tube and boiled. The samples were then loaded onto a 7.5% SDS polyacrylamide gel. Image J was used to measure the Orb protein upper:lower band ratio.

## Supporting information

S1 Fig *oskar* mRNA does not co-localize with Orb protein or mRNA in *gurken* mutants that fail to repolarize the *oocyte*. Top: Orb protein localizes around the cortex of *gurken*^*2B/2E12*^ mutant oocytes and *oskar* mRNA localizes in the middle of the oocyte. Bottom: *orb* mRNA localizes in the same pattern as Orb protein, and neither are enriched in the middle of the oocyte with *oskar* mRNA. All scale bars are 50 microns.
(DOC)

S2 Fig MT defects in *orb* mutant egg chambers are observed prior to stage 10. (A) Kinesin-βgal is enriched at the posterior in an *orb*^*mel*^ heterozygous background. (B) Kin-βgal is not enriched at the posterior in *343/mel*. (C) α-tubulin is enriched subcortically in wild type (arrows). (D) In *343/mel* α-tubulin is diffuse throughout the cytoplasm. (E) In wild type oocytes the oocyte nucleus is positioned in the dorsal anterior corner. (F) A *343/mel* egg chamber in which the oocyte nucleus has not been correctly positioned at the dorsal anterior corner. All scale bars 50 microns.
(DOC)

S3 Fig Depletion of Orb protein during mid-stages with *orb* RNAi. In wild type stage 10 oocytes, Orb protein is enriched subcortically along the oocyte cortex. When *orb* RNAi (#64002) is expressed using a midstage driver, maternal α-tubulin Gal4 (#7062), Orb protein levels are substantially reduced and the remaining Orb protein is displaced from the subcortical regions of the oocyte. The oocyte nucleus is mispositioned along the lateral cortex (arrow).
(DOC)

S4 Fig Kekkon-lacZ is expressed at the posterior of the egg chamber during early stages in *orb* mutants. (A-B) In both *orb*^*mel*^*/+* and *orb*^*343*^*/orb*^*mel*^ backgrounds *kekkon-lacZ* is expressed prior to repolarization of the oocyte in posterior follicle cells. Following repolarization, *kekkon-lacZ* is expressed in dorsal follicle cells in *orb*^*mel*^*/+*, but not in *orb*^*343/mel*^. All scale bars 10 microns.
(DOC)

S5 Fig *bicoid* mRNA localizes at the oocyte anterior in both wild type and *orb* mutants. *bicoid* mRNA is localized at the oocyte anterior at stage 10 in both wild type and *343/mel* mutant oocytes. Scale bars are 50 microns.
(DOC)

S6 Fig *cdc42* interacts with *orb* and *aPKC*, and triple heterozygotes have decreased levels of Gurken and localized *oskar* mRNA. (A and D) Staining of control ovaries expressing a fluorescent protein tagged with an NLS and (B and E) triple heterozygote (*cdc42*^*4*^*/+; aPKC*^*k06403*^*/+; orb*^*343*^*/+*) ovaries within the same tube and mounting the samples together allows for quantification of the average intensity of Gurken protein (C) and the average length of the posterior cap of *oskar* mRNA (F). Images are maximum intensity projections and scale bars are 50 microns.
(DOC)

S7 Fig Distribution of *aPKC* mRNAs in developing egg chambers. (A) Localization of *aPKC* mRNA in the germarium and young egg chamber. While *aPKC* mRNA is found in both somatic and germline cells in the germarium and in early egg chambers, the highest levels are present in the oocyte. Scale bar 10 microns. (B) During mid-stages of oogenesis, prior to oocyte repolarization, *aPKC* mRNA is distributed uniformly in nurse cells and in follicle cells, while the highest levels are localized to the oocyte. Scale bar 50 microns. (C) In stage 9 and older chambers, much of the *aPKC* mRNA is found in the nurse cells. However, within the oocyte there is an uneven distribution. A fraction of the *aPKC* mRNA in the oocyte is associated with the anterior and anterior lateral cortex (red arrowheads). Scale bar 50 microns.
(DOC)

S8 Fig Defects in Par-1 cortical enrichment correlate with actin defects in *orb^343^/orb^mel^*. (A and A’) Antibody staining for the maternally expressed GFP-Par-1-N1S transgene shows enrichment at the posterior cortex in *orb*^*mel*^ heterozygotes, and Actin is tightly organized on the oocyte cortex. (B-C) Cortical localization of GFP-Par-1 is disrupted in *343/mel* (also see Fig 6). Arrows in (B’) and (C’) point to defects in cortical Actin organization. Scale bars are 50 microns.
(DOC)

S9 Fig Shot association with the oocyte cortex depends upon *orb*. (A-A’) In wild type oocytes at stages 9–11, Shot-YFP localizes in a punctate pattern along the anterior and anterior lateral cortex (A’ arrowheads). It appears to be associated with the internal surface of the cortical actin network. (B-B’) In *orb*^*343*^*/orb*^*mel*^, the tight association of Shot-YFP with the cortical actin network is disrupted, and it shows only a diffuse association with the cortex. (A-B) Actin: red; Shot-YFP: green, scale bars 50 microns.
(DOC)

S10 Fig Patronin association with the oocyte cortex depends upon *orb*. (A-A”) In wild type and *orb*^*mel*^*/+* (shown here) stage 9–11, endogenously tagged Patronin-YFP localizes in a punctate pattern along the anterior and anterior-lateral oocyte cortex. It appears to be associated with the internal surface of the cortical actin network. (B-B”) In *orb*^*343*^*/orb*^*mel*^ the localization of Patronin-YFP along the cortex is disrupted. (A and B) Actin: red; Shot-YFP: green, scale bar 50 microns. (A’, A”, B’,B”) The anterior-lateral oocyte cortex, scale bar 10 microns. In A’ and B’, upper green line corresponds to Patronin-YFP in the follicle cells. Red line corresponds to the cortical actin network. The punctate green line in A’ and A” prime correspond to Patronin-YFP associated with the cortical actin network. In B’ and B” the association of Patronin-YFP with the cortical actin network is lost (bottom arrow). Actin: red; Patronin-YFP: green.
(DOC)

S11 Fig Orb activity depends on *aPKC*. (A) Levels of β-gal expression in HD19, *orb*^*343*^*/+* compared with protein expression levels in *aPKC*^*k06403/ex48*^. The *HD19* transgene encodes the *lacZ* sequenced fused to the *orb* 3’UTR. Levels of β-gal are decreased when *aPKC* is compromised. (B) Quantification of the ratio of β-gal:BEAF signal, normalized to the control. β-gal is reduced 2.4-fold when *aPKC* is compromised compared to the control and the p-value is less than 0.005. (C) Western showing Orb protein from wild type and *aPKC*^*k06403/ex48*^ ovaries. In wild type phosphorylated Orb protein migrates as a doublet. Proteins in the upper band are more heavily phosphorylated than those migrating in the lower band. The ratio of upper to lower Orb isoforms is altered in *aPKC* mutant ovaries. (D) Quantification of the ratio of Orb upper to lower band of in wild type compared with *aPKC* compromised ovaries. In the *aPKC* mutant ovaries the ratio is reduced compared to wild type. p-value is less than 0.05.
(DOC)

S1 Movie Cytoplasmic movements in *patronin-YFP/+; orb^mel^/+*. Cytoplasmic movements in a stage 9 *orb*^*mel*^*/+* oocyte also expressing one copy of endogenously tagged Patronin-YFP. Movie is shown at 30 frames per second. 1 frame was captured every 5 seconds for at least 2 minutes.
(MOV)

S2 Movie Premature cytoplasmic streaming in *patronin-YFP/+; orb^mel^/orb^343^*. Fast, directional cytoplasmic streaming in the oocyte of a young stage 9 *patronin-YFP/+; orb*^*mel*^*/orb*^*343*^ egg chamber. Movie is shown at 30 frames per second. 1 frame was captured every 5 seconds for at least 2 minutes.
(MOV)

S3 Movie Additional example of premature cytoplasmic streaming in *patronin-YFP/+; orb^mel^/orb^343^*. Another example of premature cytoplasmic streaming in a *patronin-YFP/+; orb*^*mel*^*/orb*^*343*^ egg chamber. Movie is shown at 30 frames per second. 1 frame was captured every 5 seconds for at least 2 minutes.
(MOV)

S1 Table Additional ventralized egg counts. Many of the genetic interactions explored in this paper were put into cups at multiple times and have a large “n” for the total number of eggs counted. Fig 4 shows only one trial in which about 400 eggs were counted. This table shows the percent ventralized eggs laid by additional genotypes such as heterozygous controls, double heterozygotes of *cdc42*^*1*^ and *orb*^*343*^ and *orb*^*343/mel*^.
(DOC)

S2 Table Primer sequences used for qPCR and oligoFISH probe sequences. (DOC)
