# Supplementary material for: The CPEB translational regulator, Orb, functions together with Par proteins to polarize the Drosophila oocyte
Source: PLoS Genet. 2019 Mar 13;15(3):e1008012. doi: 10.1371/journal.pgen.1008012 (PMC6433291; doi:10.1371/journal.pgen.1008012)
Supplement: S1 Fig — Top: Orb protein localizes around the cortex of gurken2B/2E12 mutant oocytes and oskar mRNA localizes in the middle of the oocyte. Bottom: orb mRNA localizes in the same pattern as Orb protein, and neither are enriched in the middle of the oocyte with oskar mRNA. All scale bars are 50 microns. (DOC) [file pgen.1008012.s001.doc]

**
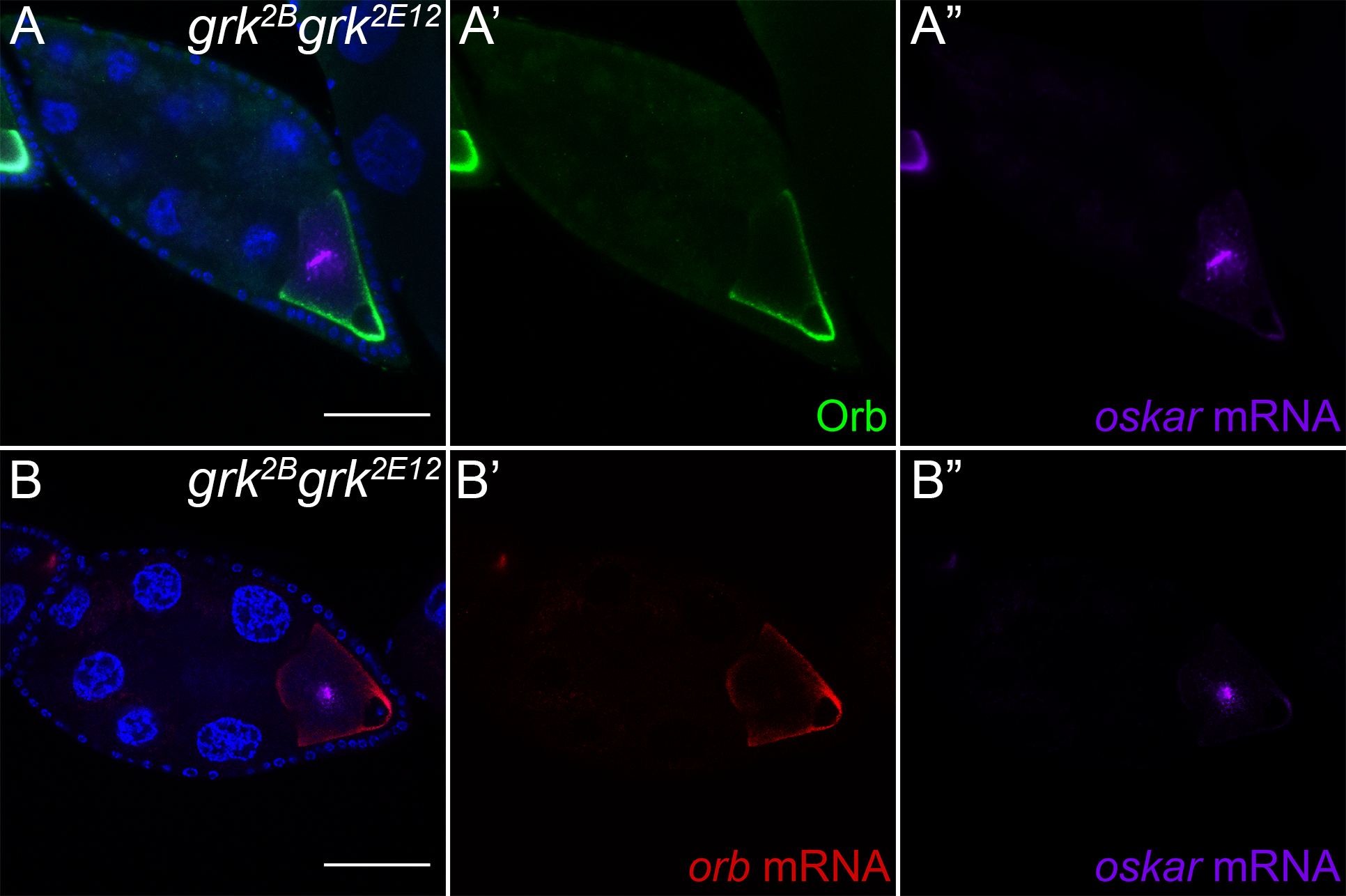
**

**S1 Fig. *oskar* mRNA does not co-localize with Orb protein or mRNA in *gurken* mutants that fail to repolarize the oocyte.**
