# Supplementary figures and images for: The CPEB translational regulator, Orb, functions together with Par proteins to polarize the Drosophila oocyte
Source: PLoS Genet. 2019 Mar 13;15(3):e1008012. doi: 10.1371/journal.pgen.1008012 (PMC6433291; doi:10.1371/journal.pgen.1008012)

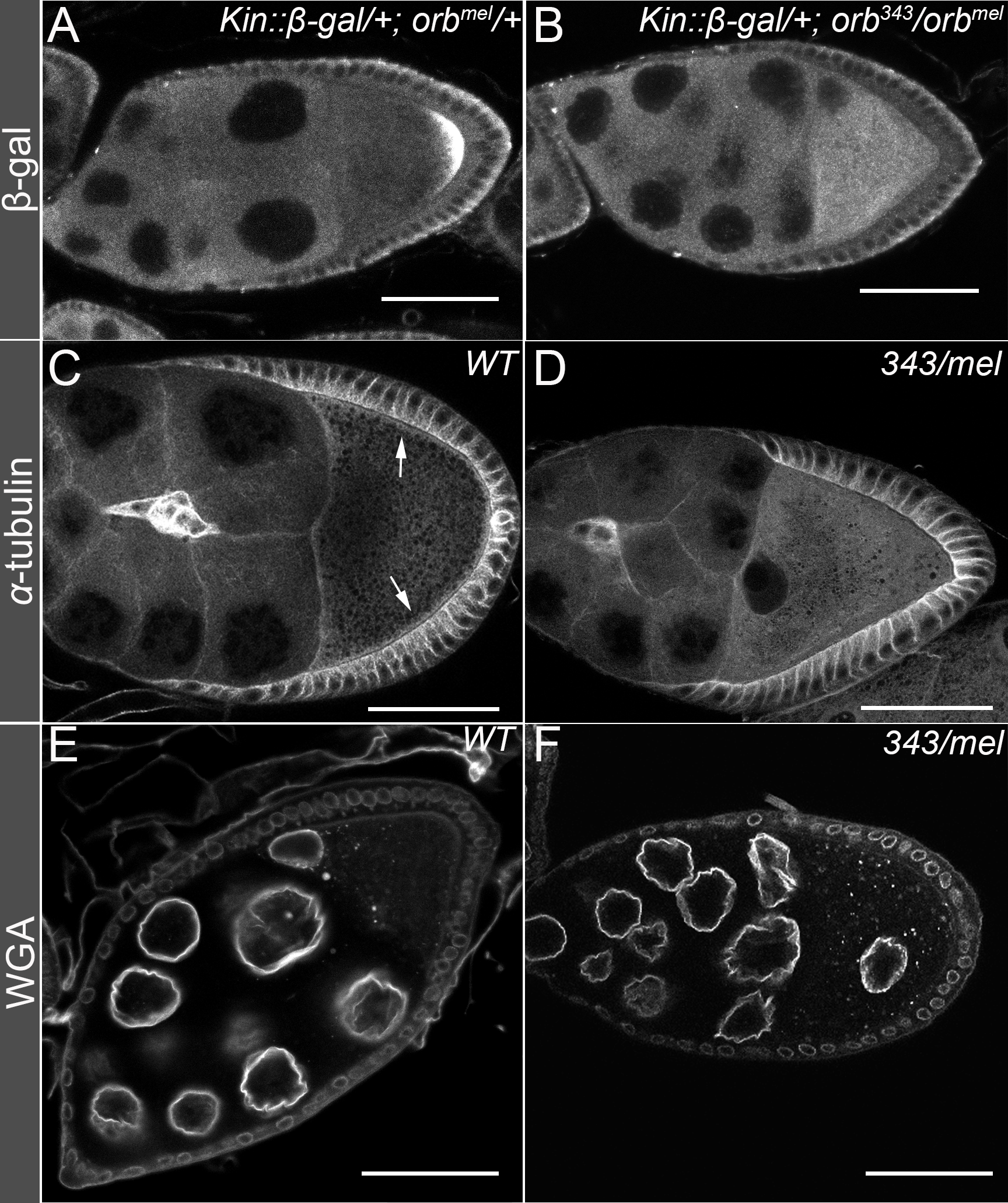


**S2 Fig. MT defects in *orb* mutant egg chambers are observed prior to stage 10**.

Supplement: S2 Fig — (A) Kinesin-βgal is enriched at the posterior in an orbmel heterozygous background. (B) Kin-βgal is not enriched at the posterior in 343/mel. (C) α-tubulin is enriched subcortically in wild type (arrows). (D) In 343/mel α-tubulin is diffuse throughout the cytoplasm. (E) In wild type oocytes the oocyte nucleus is positioned in the dorsal anterior corner. (F) A 343/mel egg chamber in which the oocyte nucleus has not been correctly positioned at the dorsal anterior corner. All scale bars 50 microns. (DOC) [file pgen.1008012.s002.doc]

**
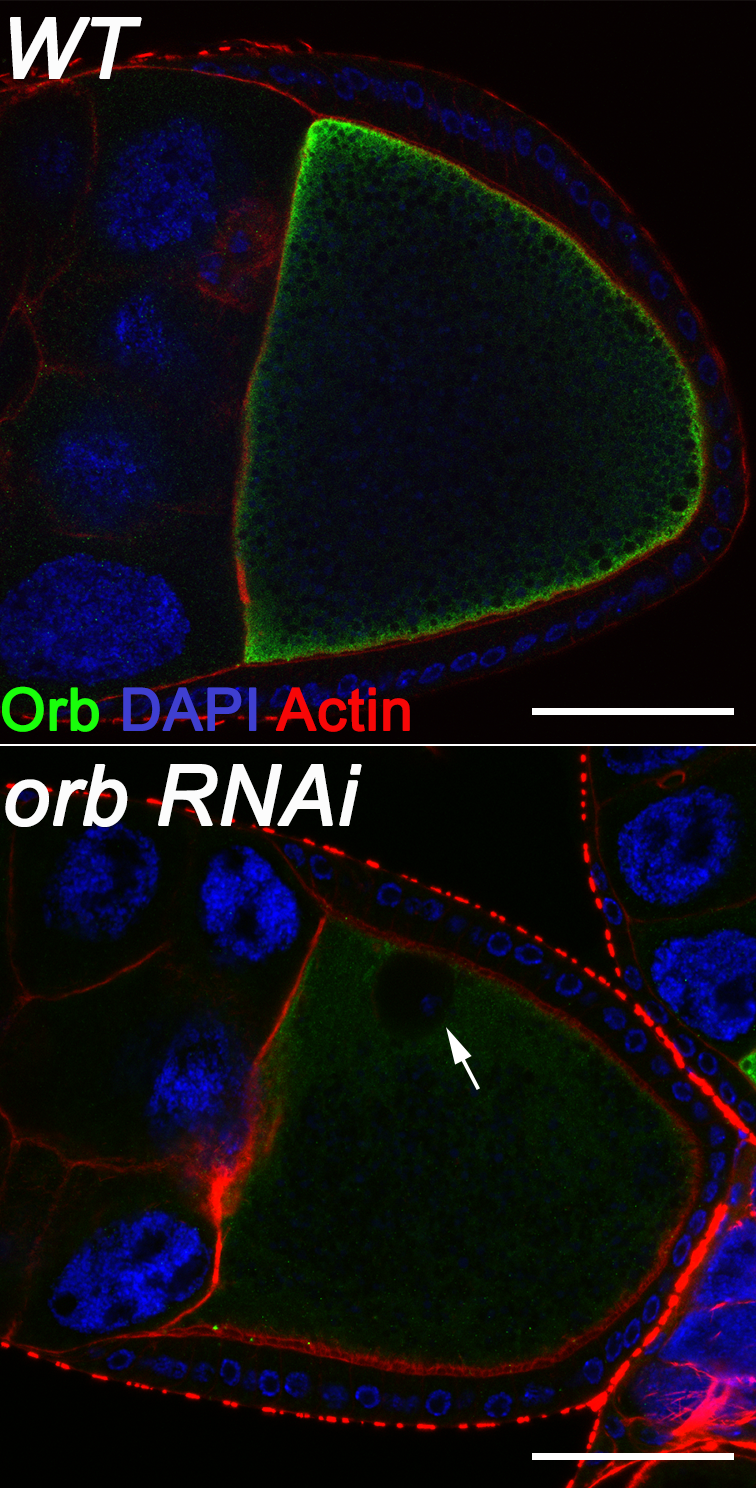
**

**S3 Fig. Depletion of Orb protein during mid-stages with *orb* RNAi.**

Supplement: S3 Fig — In wild type stage 10 oocytes, Orb protein is enriched subcortically along the oocyte cortex. When orb RNAi (#64002) is expressed using a midstage driver, maternal α-tubulin Gal4 (#7062), Orb protein levels are substantially reduced and the remaining Orb protein is displaced from the subcortical regions of the oocyte. The oocyte nucleus is mispositioned along the lateral cortex (arrow). (DOC) [file pgen.1008012.s003.doc]

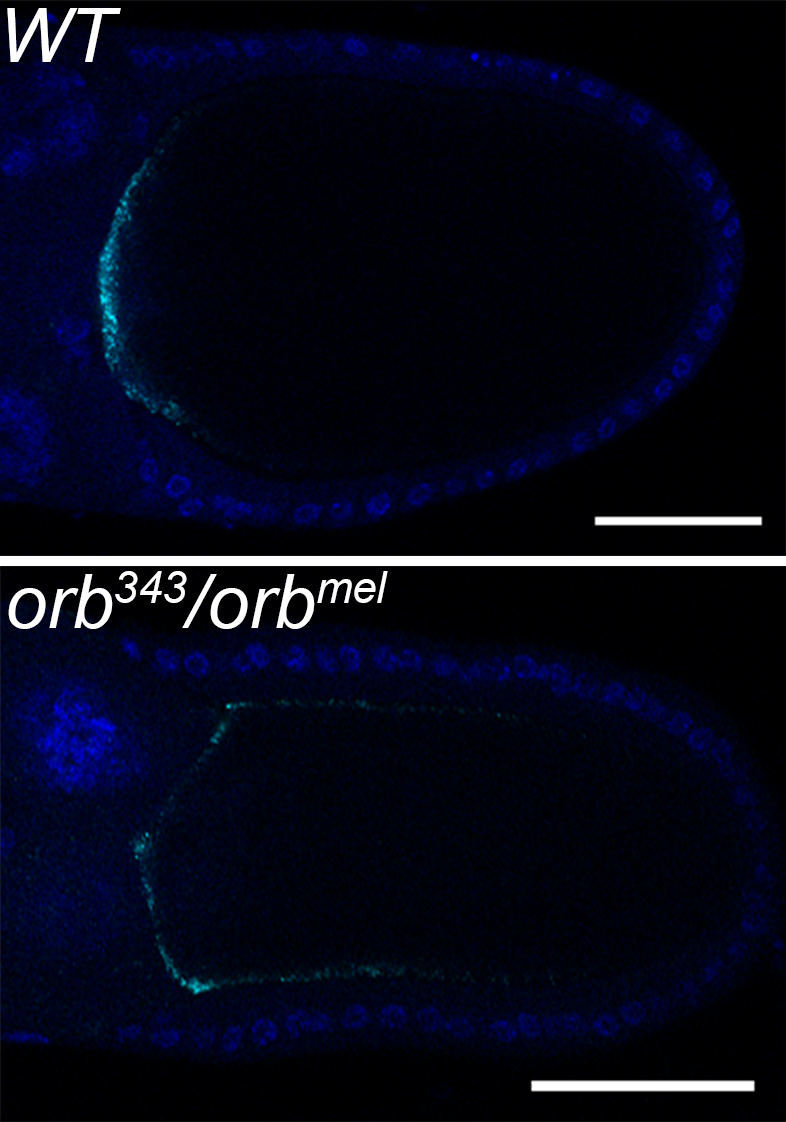


**S5 Fig. *bicoid* mRNA localizes at the oocyte anterior in both wild type and *orb*mutants.**

Supplement: S5 Fig — bicoid mRNA is localized at the oocyte anterior at stage 10 in both wild type and 343/mel mutant oocytes. Scale bars are 50 microns. (DOC) [file pgen.1008012.s005.doc]

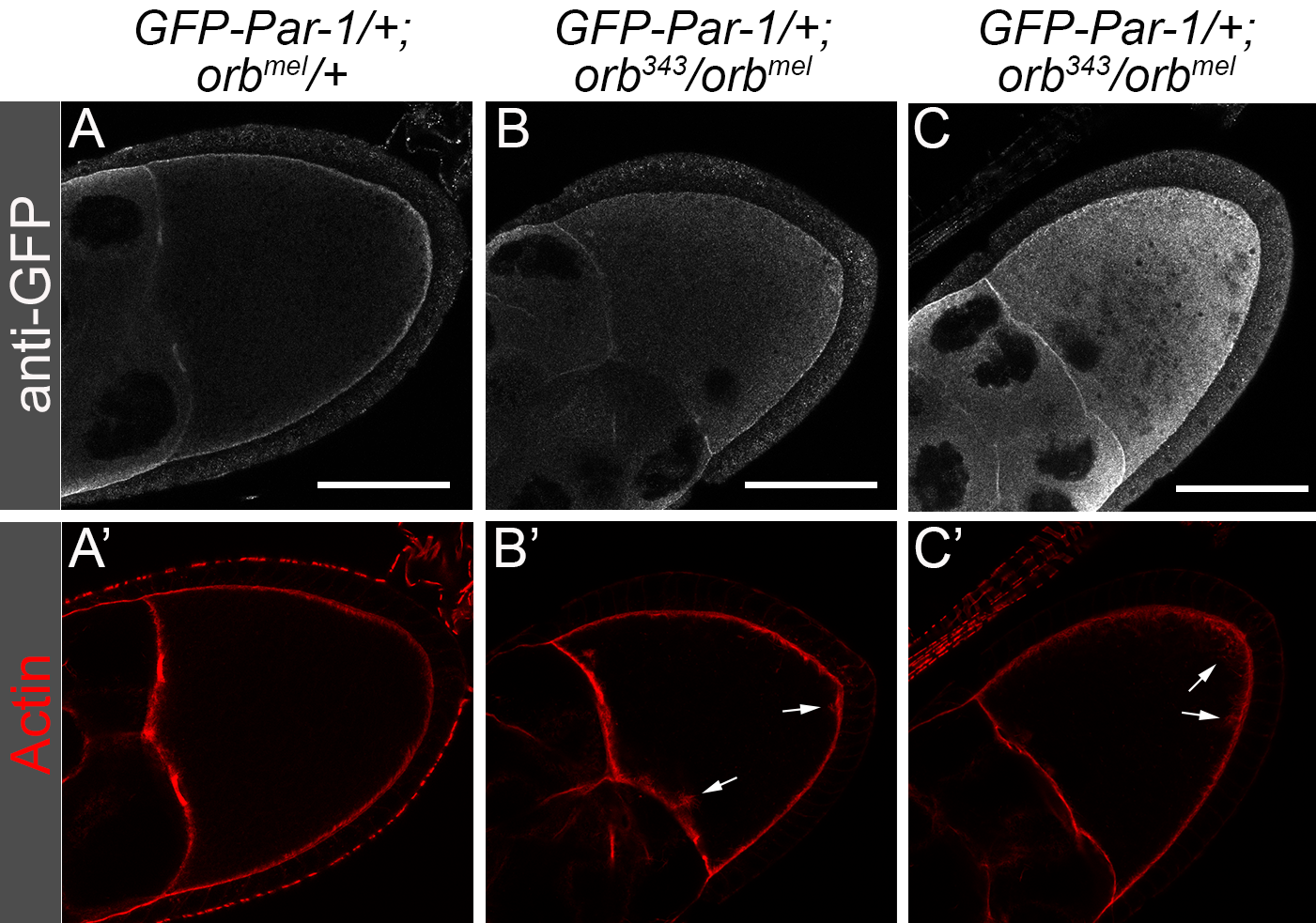


**S7 Fig. Defects in Par-1 cortical enrichment correlate with actin defects in *orb343/orbmel*.**

Supplement: S7 Fig — (A) Localization of aPKC mRNA in the germarium and young egg chamber. While aPKC mRNA is found in both somatic and germline cells in the germarium and in early egg chambers, the highest levels are present in the oocyte. Scale bar 10 microns. (B) During mid-stages of oogenesis, prior to oocyte repolarization, aPKC mRNA is distributed uniformly in nurse cells and in follicle cells, while the highest levels are localized to the oocyte. Scale bar 50 microns. (C) In stage 9 and older chambers, much of the aPKC mRNA is found in the nurse cells. However, within the oocyte there is an uneven distribution. A fraction of the aPKC mRNA in the oocyte is associated with the anterior and anterior lateral cortex (red arrowheads). Scale bar 50 microns. (DOC) [file pgen.1008012.s007.doc]

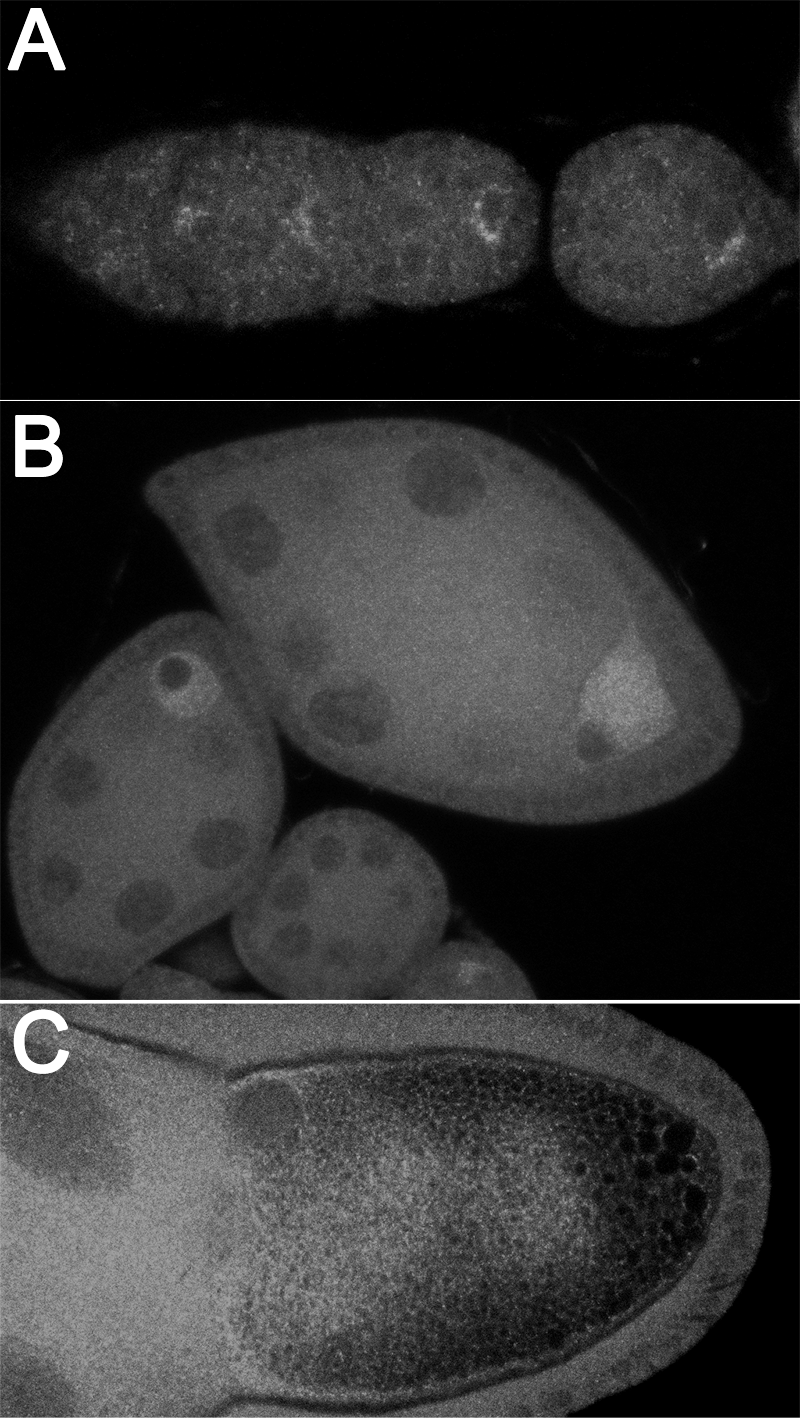


**S8 Fig. Distribution of *apkc* mRNAs in developing egg chambers.**

Supplement: S8 Fig — (A and A’) Antibody staining for the maternally expressed GFP-Par-1-N1S transgene shows enrichment at the posterior cortex in orbmel heterozygotes, and Actin is tightly organized on the oocyte cortex. (B-C) Cortical localization of GFP-Par-1 is disrupted in 343/mel (also see Fig 6). Arrows in (B’) and (C’) point to defects in cortical Actin organization. Scale bars are 50 microns. (DOC) [file pgen.1008012.s008.doc]

**
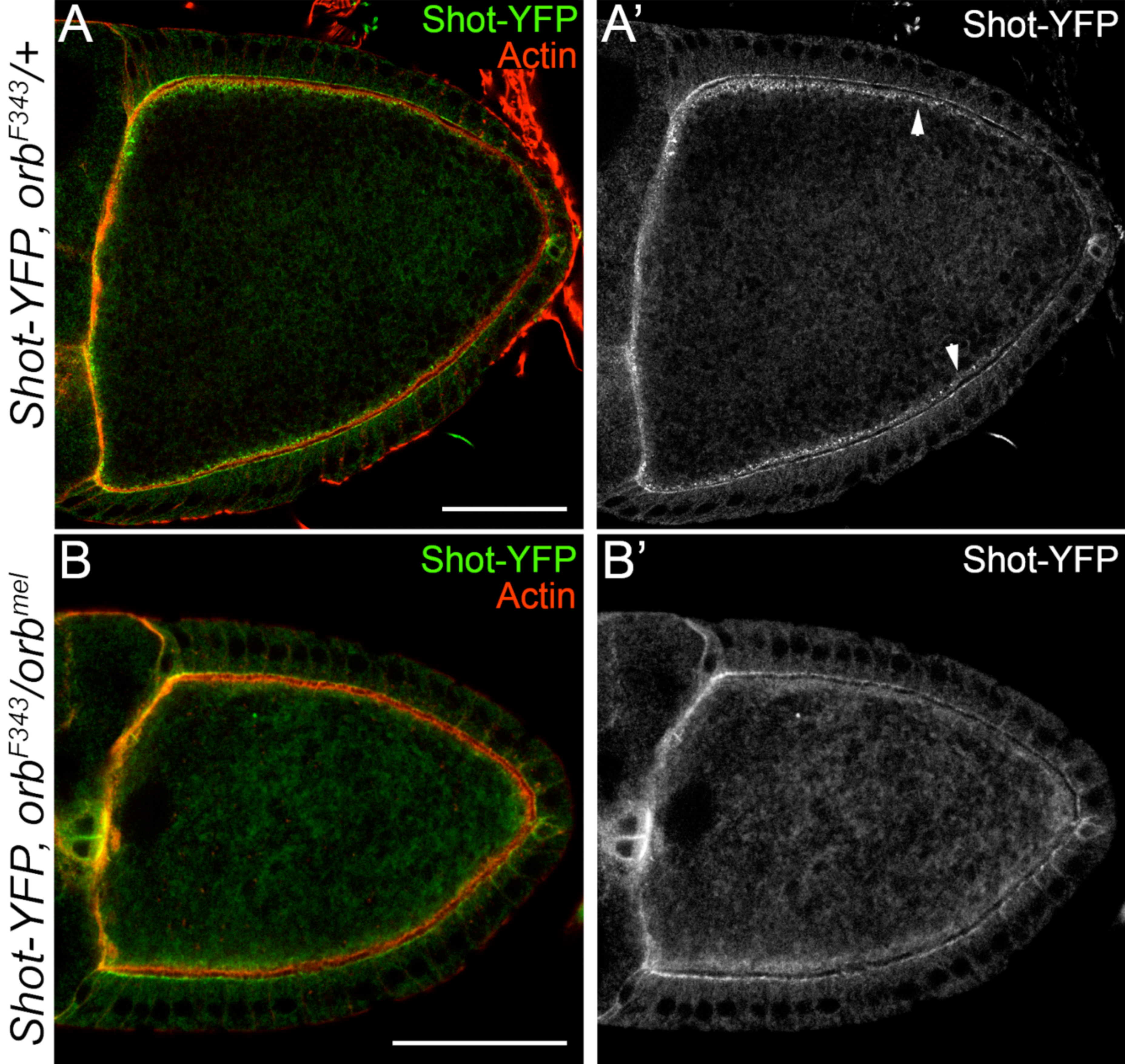
**

**S9 Fig. Shot association with the oocyte cortex depends upon *orb*.**

Supplement: S9 Fig — (A-A’) In wild type oocytes at stages 9–11, Shot-YFP localizes in a punctate pattern along the anterior and anterior lateral cortex (A’ arrowheads). It appears to be associated with the internal surface of the cortical actin network. (B-B’) In orb343/orbmel, the tight association of Shot-YFP with the cortical actin network is disrupted, and it shows only a diffuse association with the cortex. (A-B) Actin: red; Shot-YFP: green, scale bars 50 microns. (DOC) [file pgen.1008012.s009.doc]

**
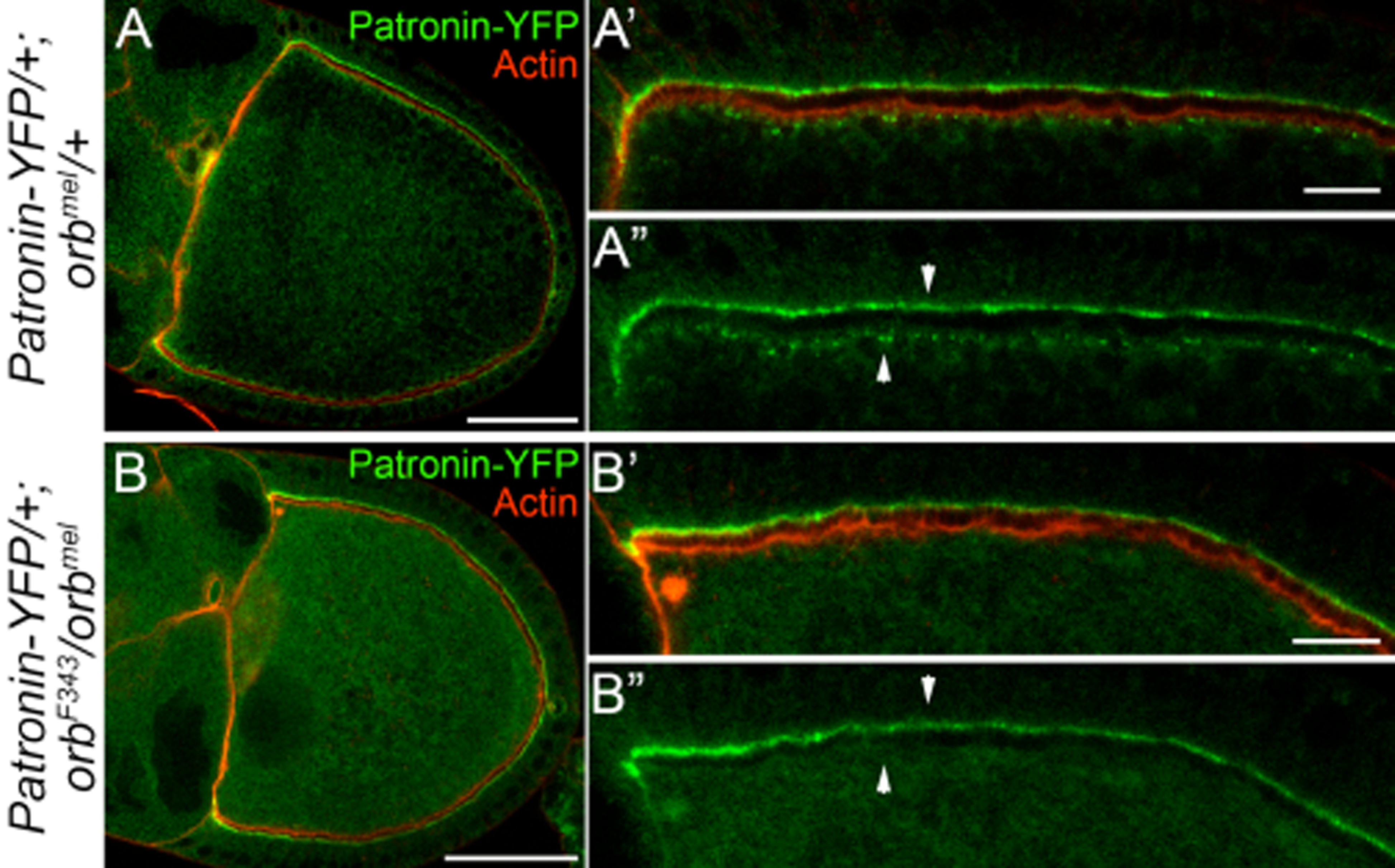
**

**S10 Fig. Patronin association with the oocyte cortex depends upon *orb*.**

Supplement: S10 Fig — (A-A”) In wild type and orbmel/+ (shown here) stage 9–11, endogenously tagged Patronin-YFP localizes in a punctate pattern along the anterior and anterior-lateral oocyte cortex. It appears to be associated with the internal surface of the cortical actin network. (B-B”) In orb343/orbmel the localization of Patronin-YFP along the cortex is disrupted. (A and B) Actin: red; Shot-YFP: green, scale bar 50 microns. (A’, A”, B’,B”) The anterior-lateral oocyte cortex, scale bar 10 microns. In A’ and B’, upper green line corresponds to Patronin-YFP in the follicle cells. Red line corresponds to the cortical actin network. The punctate green line in A’ and A” prime correspond to Patronin-YFP associated with the cortical actin network. In B’ and B” the association of Patronin-YFP with the cortical actin network is lost (bottom arrow). Actin: red; Patronin-YFP: green. (DOC) [file pgen.1008012.s010.doc]

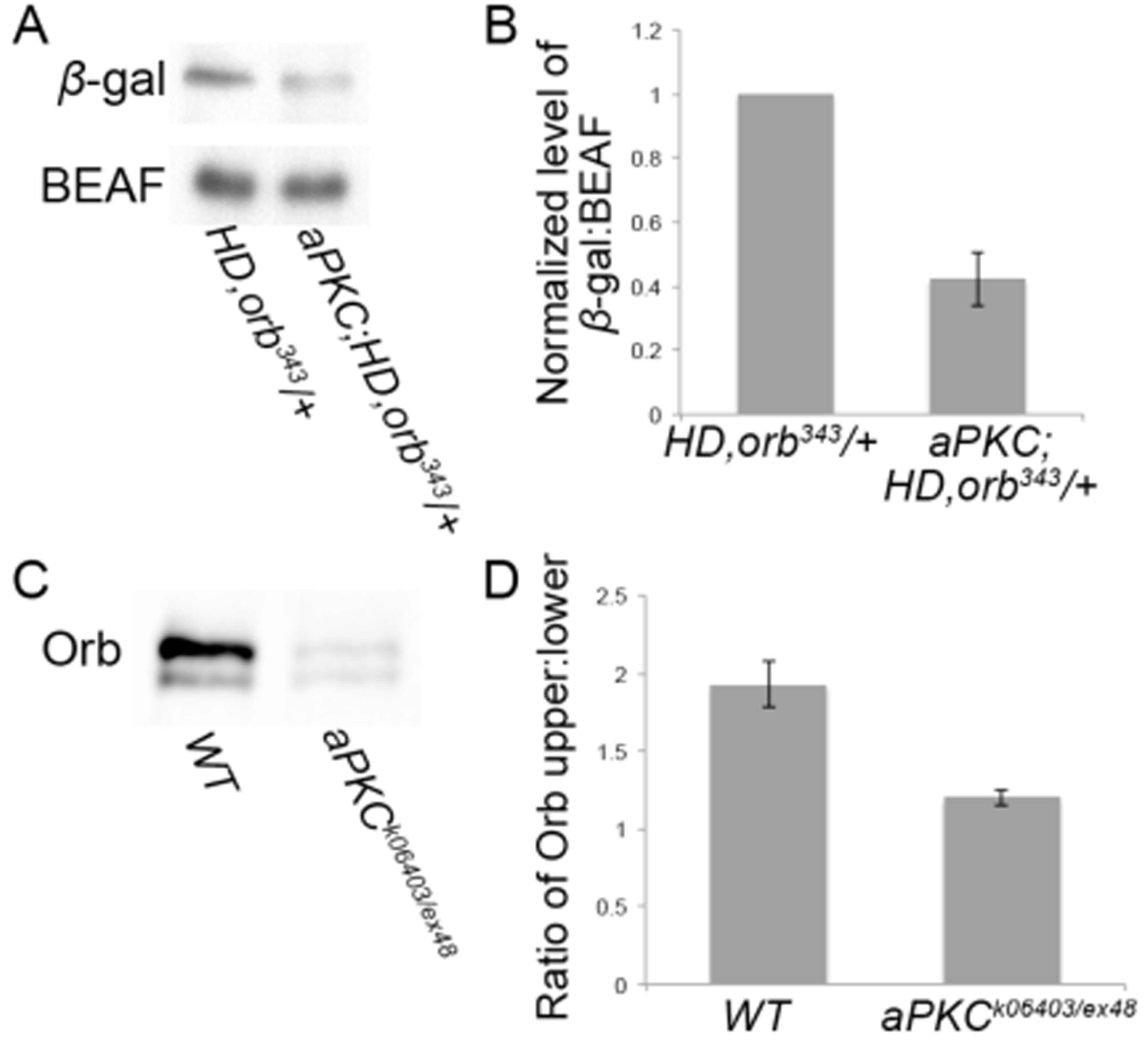


**S11 Fig. Orb activity depends on *aPKC*.**

Supplement: S11 Fig — (A) Levels of β-gal expression in HD19, orb343/+ compared with protein expression levels in aPKCk06403/ex48. The HD19 transgene encodes the lacZ sequenced fused to the orb 3’UTR. Levels of β-gal are decreased when aPKC is compromised. (B) Quantification of the ratio of β-gal:BEAF signal, normalized to the control. β-gal is reduced 2.4-fold when aPKC is compromised compared to the control and the p-value is less than 0.005. (C) Western showing Orb protein from wild type and aPKCk06403/ex48 ovaries. In wild type phosphorylated Orb protein migrates as a doublet. Proteins in the upper band are more heavily phosphorylated than those migrating in the lower band. The ratio of upper to lower Orb isoforms is altered in aPKC mutant ovaries. (D) Quantification of the ratio of Orb upper to lower band of in wild type compared with aPKC compromised ovaries. In the aPKC mutant ovaries the ratio is reduced compared to wild type. p-value is less than 0.05. (DOC) [file pgen.1008012.s011.doc]

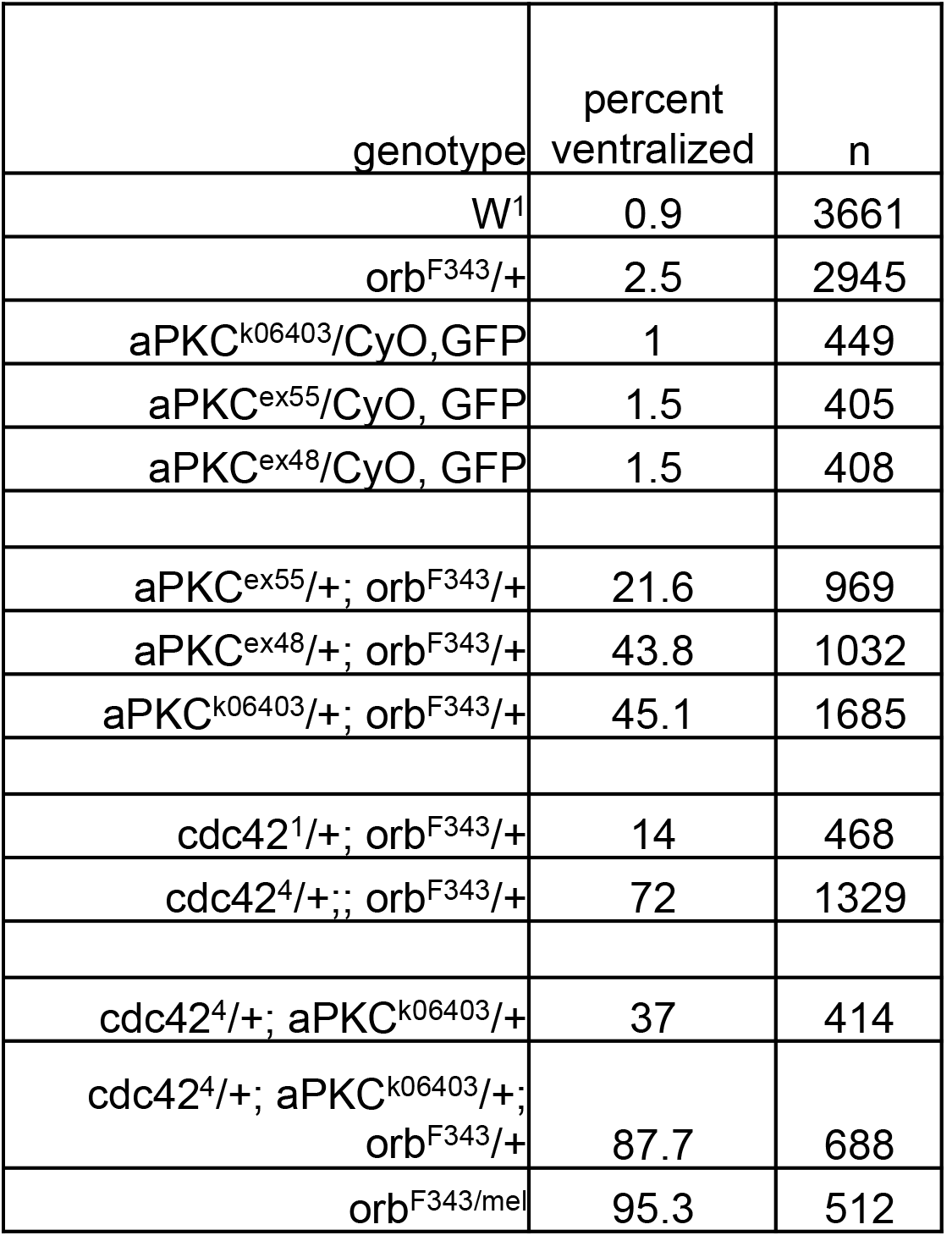


**S1 Table. Additional ventralized egg counts.**

Supplement: S1 Table — Many of the genetic interactions explored in this paper were put into cups at multiple times and have a large “n” for the total number of eggs counted. Fig 4 shows only one trial in which about 400 eggs were counted. This table shows the percent ventralized eggs laid by additional genotypes such as heterozygous controls, double heterozygotes of cdc421 and orb343 and orb343/mel. (DOC) [file pgen.1008012.s015.doc]

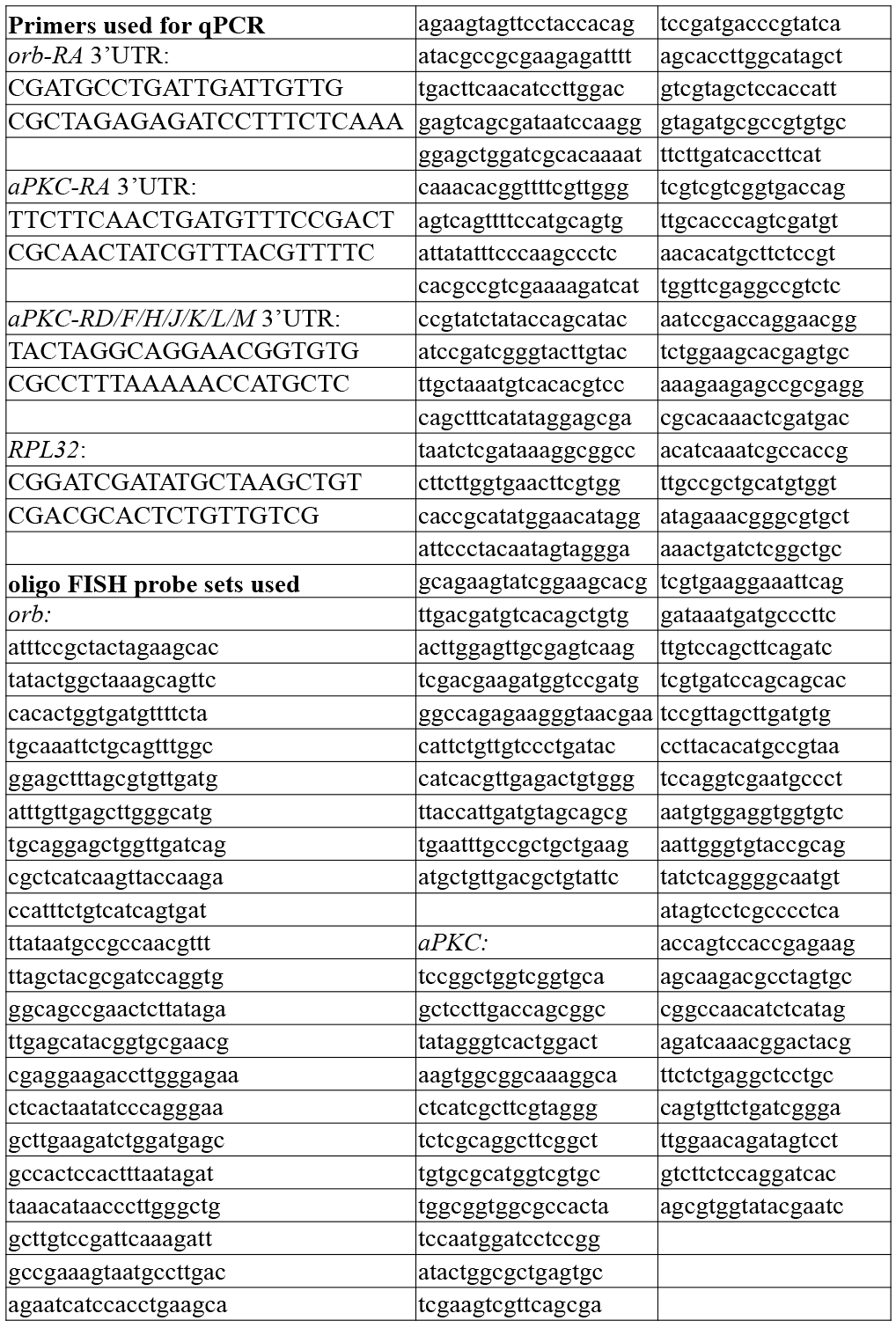


**S2 Table. Primer sequences used for qPCR and oligoFISH probe sequences.**

Supplement: S2 Table — (DOC) [file pgen.1008012.s016.doc]
