# Supplementary material for: The CPEB translational regulator, Orb, functions together with Par proteins to polarize the Drosophila oocyte
Source: PLoS Genet. 2019 Mar 13;15(3):e1008012. doi: 10.1371/journal.pgen.1008012 (PMC6433291; doi:10.1371/journal.pgen.1008012)
Supplement: S4 Fig — (A-B) In both orbmel/+ and orb343/orbmel backgrounds kekkon-lacZ is expressed prior to repolarization of the oocyte in posterior follicle cells. Following repolarization, kekkon-lacZ is expressed in dorsal follicle cells in orbmel/+, but not in orb343/mel. All scale bars 10 microns. (DOC) [file pgen.1008012.s004.doc]

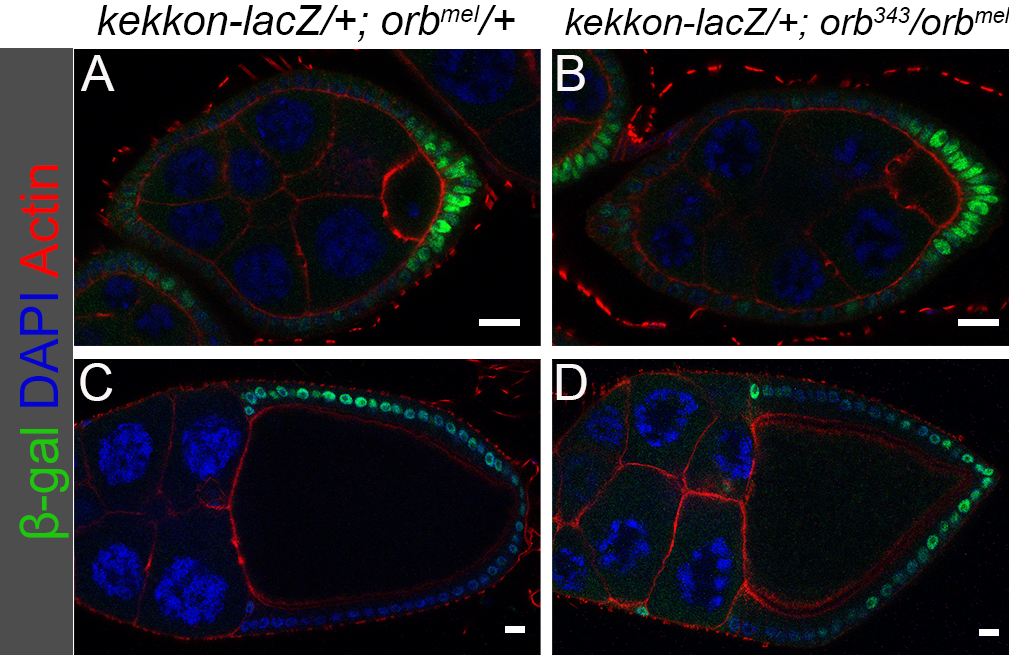


**S4 Fig. Kekkon-lacZ is expressed at the posterior of the egg chamber during early stages in *orb* mutants.**
