# Supplementary material for: The CPEB translational regulator, Orb, functions together with Par proteins to polarize the Drosophila oocyte
Source: PLoS Genet. 2019 Mar 13;15(3):e1008012. doi: 10.1371/journal.pgen.1008012 (PMC6433291; doi:10.1371/journal.pgen.1008012)
Supplement: S6 Fig — (A and D) Staining of control ovaries expressing a fluorescent protein tagged with an NLS and (B and E) triple heterozygote (cdc424/+; aPKCk06403/+; orb343/+) ovaries within the same tube and mounting the samples together allows for quantification of the average intensity of Gurken protein (C) and the average length of the posterior cap of oskar mRNA (F). Images are maximum intensity projections and scale bars are 50 microns. (DOC) [file pgen.1008012.s006.doc]

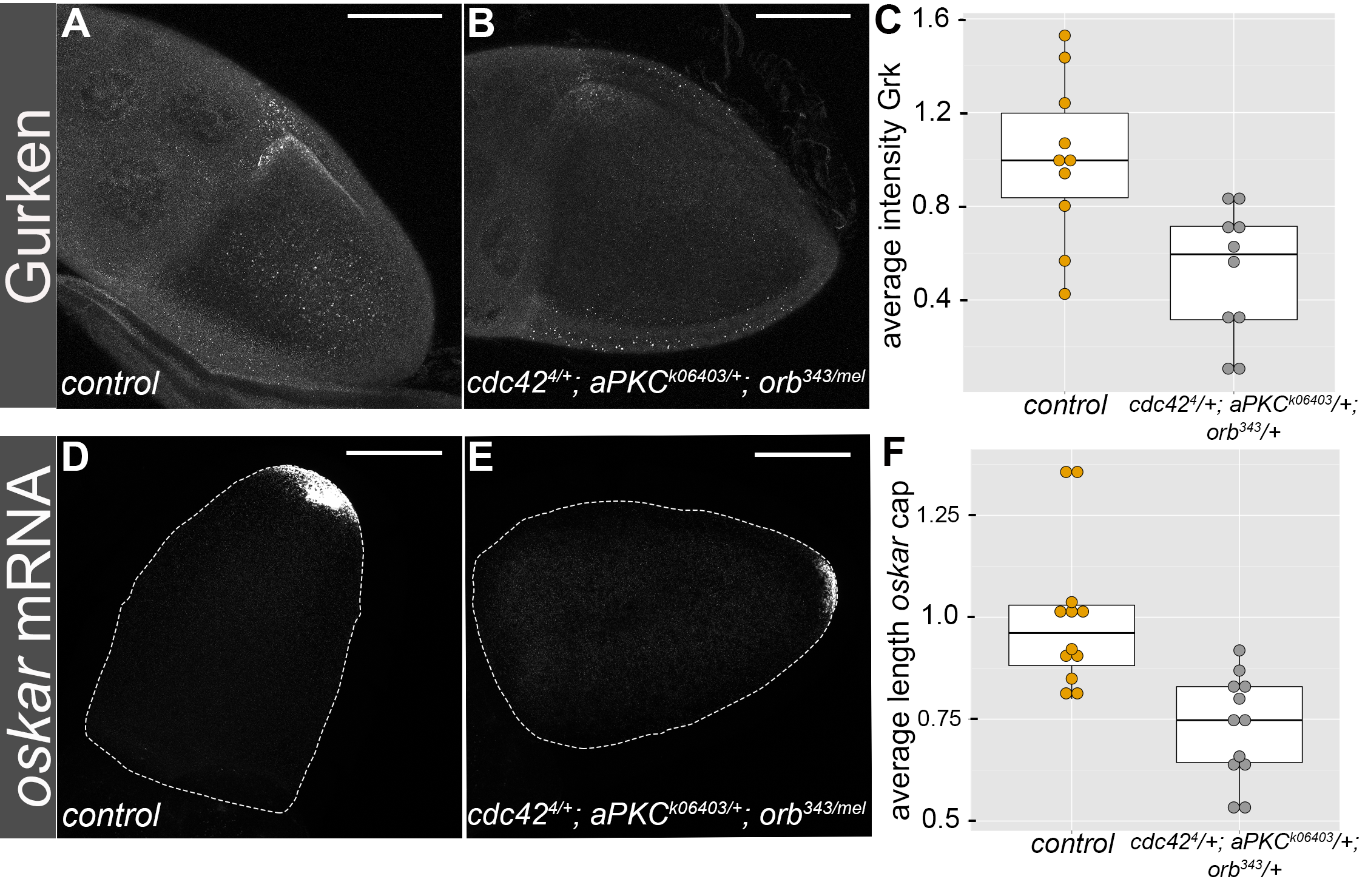


**S6 Fig. *cdc42* interacts with *orb* and *aPKC*, and triple heterozygotes have decreased levels of Gurken and localized *oskar* mRNA*.***
